# Transcriptome Analysis and Identification of Chemosensory Genes in *Leguminivora glycinivorella*

**DOI:** 10.3390/biology15060505

**Published:** 2026-03-21

**Authors:** Jiaqi Shi, Yuxin Zhou, Zhengxiao Du, Ruirui Li, Qi Wang, Yu Gao, Shusen Shi

**Affiliations:** 1 Key Laboratory of Soybean Disease and Pest Control, Ministry of Agriculture and Rural Affairs, College of Plant Protection, Jilin Agricultural University, Changchun 130118, China; shijiaqi0517@163.com (J.S.); wisdomking@foxmail.com (Z.D.); liruirui2026@163.com (R.L.); wq980828@126.com (Q.W.); gaothrips@jlau.edu.cn (Y.G.); 2 State Key Laboratory of Green Pesticide, Guizhou University, Guiyang 550025, China

**Keywords:** *Leguminivora glycinivorella*, Olethreutidae, chemosensory genes, transcriptome

## Abstract

The soybean pod borer, *Leguminivora glycinivorella*, is a monophagous pest that threatens soybean production. Its larvae feed concealed within pods, which limits the efficacy of conventional insecticides. Elucidating its chemosensory system is therefore essential for developing green, behavior-based management strategies. Reference-based transcriptomics across multiple tissues of *L. glycinivorella* identified a comprehensive repertoire of chemosensory genes, including 76 odorant receptors (ORs), 15 gustatory receptors (GRs), 18 ionotropic receptors (IRs), 52 odorant-binding proteins (OBPs), 18 chemosensory proteins (CSPs), and 4 sensory neuron membrane proteins (SNMPs). Sequence and phylogenetic analyses characterized these candidates within the context of known insect chemosensory families. Notably, canonical bitter GRs and specific IR lineages (e.g., IR100/IR85a) were not detected in our dataset, potentially reflecting adaptation to the specialized soybean-feeding habit of this pest. Expression profiling further revealed pronounced sexual and tissue dimorphism: male antennae showed significant enrichment of putative pheromone receptors (PRs) and *LglySNMP1*, whereas several OBPs and ORs exhibited female-biased expression, suggesting roles in host location and oviposition. Additionally, the high expression of GR43a homologs points to fructose sensing, while the lack of detectable CO_2_ receptor components (except *LglyGR2*) suggests atypical carbon dioxide perception mechanisms. Collectively, this study provides a valuable expression atlas of chemosensory genes in *L. glycinivorella* and identifies sex-specific candidate genes for future functional validation and behavior-based pest management.

## Simple Summary

The soybean pod borer is a serious soybean pest because its larvae feed inside pods and damage developing seeds, making conventional insecticide control difficult. To support the development of safer and more effective management strategies, we investigated the genes that may help this insect detect chemical cues from host plants, mates, and its surroundings. By analyzing transcriptome data from multiple adult tissues, we identified 183 candidate genes related to chemical sensing. Many of these genes showed clear differences in expression among tissues and between females and males. In particular, genes associated with odor detection were mainly enriched in the antennae, the primary sensory organs for smelling. Male antennae showed high expression of several candidate genes that may be involved in pheromone detection, whereas some genes were more highly expressed in females and may contribute to host finding and egg-laying site selection. We also found that some expected sensory gene groups were absent from the dataset, which may be related to the insect’s close adaptation to soybean. These results provide a foundation for future functional studies and may help support environmentally friendly pest control based on chemical communication.

## 1. Introduction

The soybean pod borer, *L. glycinivorella* (Matsumura, 1898), a member of the family Olethreutidae (Lepidoptera), is a univoltine, highly host-specific pest that overwinters as mature larvae in the soil [1]. It is widely distributed across China, Japan, and other parts of East Asia, and ranks as one of the most destructive pests of soybean in northeastern and northern China [1]. Larvae bore into pods and feed on developing seeds, causing significant yield losses (typically 10–20%, up to >40% in severe outbreaks) and reducing grain quality and market value; infestation levels have been reported to increase in some regions/years [2]. Because larvae feed inside sealed pods, conventional chemical insecticides cannot effectively reach them, posing major challenges for control [3]. Consequently, research has increasingly shifted toward chemical signaling, and deciphering the molecular mechanisms underlying insect chemoperception has become an important avenue for developing novel green pest management strategies. Recent work has advanced our understanding of *L. glycinivorella*’s diapause regulation [4], transgenic resistance [5], microbial control [6], and biological control [7,8] and even identified new associated pests like *Orosius orientalis* (Matsumura, 1914) [9]. However, despite these efforts, the molecular basis of its olfactory system, which is central to host finding, mate location, and oviposition, remains uncharacterized. To date, no comprehensive study has identified or profiled its chemosensory receptor genes, leaving a critical gap in the development of behavior-based management tools.

In recent years, research on insect chemosensory systems has emerged as a focal point at the intersection of chemical ecology, behavioral biology, and sustainable pest control [10,11]. Insects rely on highly specialized chemosensory mechanisms to detect complex environmental cues—such as plant volatile organic compounds (VOCs), sex pheromones, oviposition stimulants, and defensive secondary metabolites [10]. This process is mediated by several conserved gene families: odorant receptors (ORs), gustatory receptors (GRs), ionotropic receptors (IRs), odorant-binding proteins (OBPs), chemosensory proteins (CSPs), and sensory neuron membrane proteins (SNMPs) [10,11,12,13,14].

Specifically, ORs are seven-transmembrane proteins that function as ligand-gated cation channels through heteromeric complex formation with the conserved co-receptor Orco. Ligand binding to plant terpenes or pheromone components triggers channel opening and the rapid activation of olfactory neurons [15,16]. GRs share a similar seven-transmembrane topology but are evolutionarily distinct from ORs—some evidence suggests that ORs may have originated from GRs. GRs primarily detect non-volatile compounds (e.g., sugars and bitter substances) via contact chemosensation and also mediate CO_2_ perception; they are abundantly expressed in mouthparts and are crucial for host recognition and feeding decisions [17]. In Lepidoptera, IRs are classified into three subfamilies: antennal IRs (A-IRs), lepidopteran-specific IRs (LS-IRs), and divergent IRs (D-IRs) [18]. A-IRs, expressed in antennae, detect specific odorants such as acids and amines [19,20,21,22,23] and also contribute to thermosensation and hygrosensation [24,25,26,27]. D-IRs, found in gustatory tissues but not antennae, are implicated in taste reception [28,29,30,31,32], while LS-IR functions remain unclear. Functional IR complexes often require co-receptors IR8a or IR25a. For example, in *Bactrocera dorsalis* (Hendel, 1912), CRISPR/Cas9 knockout of IR8a impairs acetic acid detection, and calcium imaging shows that IR64a and IR75a (paired with IR76b or IR8a) act as candidate acid receptors, potentially complementing detection channels mediated by ORs/GRs [31,32].

Beyond membrane receptors, soluble carrier proteins—OBPs and CSPs—play essential roles in transporting hydrophobic odorants through aqueous sensillum lymph to receptors. OBPs typically contain six conserved cysteines and exhibit selectivity for odor molecules, accumulating in antennal lymph [33]. Although the functional necessity of OBPs has been questioned in some insect models, they are widely regarded as critically important for olfactory perception in Lepidoptera Odorant Reception in Insects: Functional and Evolutionary Perspectives. CSPs, with only four conserved cysteines, possess more compact, thermostable structures and broader expression—not only in olfactory organs but also in wings, legs, and hemolymph—suggesting roles beyond olfaction, such as in development or immunity [34]. SNMPs, CD36-family transmembrane glycoproteins localized on olfactory neuron membranes, enhance sensitivity to sex pheromones by interacting with pheromone receptors; loss of SNMP1 can severely disrupt mating behavior [35,36].

Notably, these chemosensory genes display pronounced tissue specificity and sexual dimorphism. For instance, in *Helicoverpa armigera* (Hübner, 1808), *HarmSNMP1* is highly expressed in antennae and is critical for pheromone detection, while *HarmGR9*—abdomen-enriched—regulates satiety and sugar intake [17]. In *Spodoptera littoralis* (Boisduval, 1833), SlitIR87a is antenna-specific, implicating it in odor sensing [31]; in *S. frugiperda*, *SfruCSP1/2* are highly expressed in larval cuticle and may serve as targets for insecticides like chlorfenapyr [37]. In *H. assulta* and *H. armigera*, mouthpart-biased CSP4 facilitates sugar feeding by reducing surface tension [36]. Moreover, in *Holotrichia parallela*, the pheromone receptor OR14 shows male-biased, circadian-rhythmic expression synchronized with its every-other-day pheromone release behavior [38]. Collectively, these findings reveal that chemosensory genes are deeply integrated into the behavioral regulatory networks governing the insect life cycle [35,39], and their sex- and tissue-biased expression provides key clues for dissecting behaviors like female oviposition preference or male pheromone tracking.

Despite extensive characterization in other major lepidopteran pests, the molecular landscape of the soybean pod borer’s chemosensory system remains unexplored. Current knowledge is limited to the identification of its sex pheromones—primarily (E)-10-dodecen-1-yl acetate and (E,E)-8,10-dodecadien-1-yl acetate—and associated electrophysiological responses (e.g., EAG and GC–EAD) [9]. However, the molecular receptors and binding proteins involved in detecting these pheromones and host volatiles have not been systematically identified, especially those in the GR and IR families.

To address this gap, we performed transcriptome sequencing of multiple adult tissues of *L. glycinivorella*, including male and female antennae, heads without antennae, thoraces, abdomens, and legs. We comprehensively annotated all six core chemosensory gene families (ORs, GRs, IRs, OBPs, CSPs, and SNMPs), analyzed their phylogenetic relationships with homologs from other Lepidoptera, and integrated tissue-specific expression profiles to prioritize candidate genes potentially involved in (1) sex pheromone perception (e.g., ORs, OBPs, and SNMPs), (2) host volatile detection (e.g., ORs and IRs), and (3) contact-mediated oviposition behavior (e.g., GRs, IRs, and CSPs). This study fills a critical gap in the molecular understanding of *L. glycinivorella* chemoreception and provides a foundational resource and promising molecular targets for future functional validation and the development of chemosensory-based green control strategies, such as behavioral disruptors, pheromone synergists, or RNA-targeted biopesticides.

## 2. Materials and Methods

### 2.1. Insect Source and Tissue Sampling

The *L. glycinivorella* colony used in this study was maintained in the insectary of the Economic Crop Pest Management and Control Team, Jilin Agricultural University. The colony was originally established from individuals collected at the Xidi experimental field in Changchun, Jilin Province, China (43.81061° N, 125.40368° E). Insects were reared for multiple generations under controlled laboratory conditions at 25 ± 1 °C, 65 ± 5% relative humidity, and a 16 h light:8 h dark photoperiod. Newly emerged adults were supplied with 10% (*v*/*v*) honey solution.

To obtain chemosensory-related transcripts from different adult tissues, eight tissue types were dissected from 3-day-old female and male adults, including female antennae (FA), male antennae (MA), female heads without antennae (FH), male heads without antennae (MH), mixed thoraces (T), mixed legs (L), female abdomens (FAb), and male abdomens (MAb). All dissections were performed on ice. Collected tissues were immediately frozen in liquid nitrogen and stored at −80 °C until RNA extraction.

### 2.2. RNA Extraction and Transcriptome Sequencing

Total RNA was extracted from each tissue using TRIzol reagent (Invitrogen, Thermo Fisher Scientific, Waltham, MA, USA). RNA purity and concentration were assessed using a NanoDrop 2000 spectrophotometer (Thermo Scientific, Thermo Fisher Scientific, Waltham, MA, USA), and RNA integrity was verified with an Agilent 2100 Bioanalyzer (Agilent Technologies, Santa Clara, CA, USA) or LabChip GX system (Revvity, Hopkinton, MA, USA). Qualified RNA samples (RIN ≥ 7.0) were sent to Biomarker Technologies (Beijing, China) for library construction and sequencing. Stranded cDNA libraries were prepared using oligo (dT) magnetic beads to enrich poly(A)+ mRNA. The mRNA was fragmented, reverse-transcribed into first- and second-strand cDNA, and then subjected to end repair, A-tailing, and adapter ligation. Libraries were size-selected using AMPure XP beads (Beckman Coulter, Brea, CA, USA), followed by PCR amplification. Library quality was assessed using a Qubit 3.0 fluorometer (Invitrogen, Thermo Fisher Scientific, Waltham, MA, USA), and libraries with concentrations ≥ 1 ng/μL were retained. Insert size distribution was validated on a Qsep400 system (BiOptic Inc., New Taipei City, Taiwan), and effective library concentration (>2 nM) was determined by qPCR. Qualified libraries were sequenced on an Illumina platform in paired-end 150 bp (PE150) mode.The raw transcriptome sequencing data generated in this study have been deposited in the NCBI Sequence Read Archive (SRA) under BioProject accession number PRJNA1434332. The corresponding BioSample and SRA run accession numbers are provided in Supplementary Table S1.

### 2.3. Transcriptome Assembly and Functional Annotation

Raw reads were quality-filtered to remove adapter-contaminated and low-quality reads, yielding clean data. Clean reads were aligned to the *L. glycinivorella* reference genome (*Leguminivora_glycinivorella*.GCF_023078275.1.genome.fa) using HISAT2 v2.0.4 [40]. Transcriptome reconstruction and expression quantification were performed using StringTie v2.2.1 [41], and gene expression levels were normalized as FPKM. Functional annotation was conducted using the BMKCloud (Biomarker Technologies) standard pipeline, including DIAMOND searches against Nr/Swiss-Prot/KEGG and related databases [42], GO assignment using InterProScan [43], and conserved domain identification using Pfam [44].

### 2.4. Identification and Structural Prediction of Chemosensory Genes

Six chemosensory gene families—OBP, CSP, OR, IR, GR, and SNMP—were identified from the annotated transcriptome. To improve annotation accuracy, homologous sequences from closely related lepidopterans were used as references, and candidate genes were confirmed by BLASTP searches against the NCBI database. Open reading frames (ORFs) were predicted using (NCBI, https://www.ncbi.nlm.nih.gov/orffinder/, accessed on 13 March 2026). Signal peptides were predicted using SignalP 5.0 (https://services.healthtech.dtu.dk/service.php?SignalP-5.0, accessed on 13 March 2026), and transmembrane domains were predicted using the TOPCONS web server (https://topcons.cbr.su.se/, accessed on 13 March 2026). Molecular weight and isoelectric point were calculated using the ExPASy Compute pI/Mw tool (https://web.expasy.org/compute_pi/, accessed on 13 March 2026). Amino acid sequence alignment and manual editing were performed using DNAMAN 8.0.

### 2.5. Phylogenetic Analysis

Amino acid sequences of *L. glycinivorella* chemosensory genes and homologous sequences from representative insect species were aligned using MAFFT v7.52 [45]. Poorly aligned and highly gapped regions were trimmed prior to phylogenetic reconstruction. Maximum-likelihood (ML) phylogenetic trees were constructed using IQ-TREE v1.6.12 [46]. The best-fit amino acid substitution model for each dataset was automatically selected using ModelFinder implemented in IQ-TREE [47], with JTT + F + R7 for ORs, VT + F + G4 for GRs, JTT + F + R8 for IRs, LG + R4 for OBPs, LG + F + R4 for CSPs, and LG + I + G4 for SNMPs. Node support was assessed using 10,000 ultrafast bootstrap replicates [48]. Because these chemosensory gene families are highly divergent and suitable outgroups were not available for reliable rooting, the ML phylogenetic trees were presented and interpreted as unrooted trees.

To further evaluate the robustness of the phylogenetic inference, Bayesian inference (BI) analyses were additionally performed based on CDS datasets. CDS sequences were aligned using MACSE v2.06 [49], and the alignments were manually inspected and trimmed in MEGA 11 [50]. BI trees were reconstructed using MrBayes 3.2.7a [51] implemented in PhyloSuite v1.2.2 [52], and the resulting trees are provided in the Supplementary Materials. Final tree visualization and annotation were performed using iTOL v7.6 [53].

### 2.6. Differential Expression Analysis

To characterize tissue-specific expression patterns, FPKM values of candidate chemosensory genes were log_2_(FPKM + 1) transformed and normalized. The processed data were uploaded to Biomarker’s online platform for heatmap visualization using default clustering parameters.

Differential expression analysis was conducted on Biomarker’s online platform using the EBSeq package. Genes with |log_2_(fold change)| > 1 and *p* < 0.05 were considered significantly differentially expressed. Special emphasis was placed on the comparison between FA and MA to identify female antenna-biased chemosensory candidates.

### 2.7. qRT-PCR Validation

To support transcriptome-derived expression profiling, qRT-PCR was performed for ten representative odorant receptor genes (*LglyORco*, *LglyOR1a*, *LglyOR11b*, *LglyOR49b*, *LglyOR6c*, *LglyOR6d*, *LglyOR15a*, *LglyOR2*, *LglyOR6b*, and *LglyOR6a*) using an independent batch of male antennae from *L. glycinivorella*. Total RNA from the independent batch of male antennae was treated with DNase I and reverse-transcribed into cDNA. qRT-PCR was performed using PerfectStart^®^ Green qPCR SuperMix (TransGen Biotech, Beijing, China; AQ601-02) according to the manufacturer’s instructions, and amplification specificity was confirmed by melting-curve analysis. Relative expression levels were calculated using the 2^−ΔΔCt^ method with Actin as the internal reference gene. Data are presented as mean ± SEM from technical replicates (wells, *n* = 9). Primer sequences are provided in Table S2.

## 3. Results

### 3.1. Transcriptome Sequencing and Assembly Quality Assessment

High-throughput transcriptome sequencing was performed on eight tissue samples of *L. glycinivorella*, including female antennae (FA), male antennae (MA), female heads without antennae (FH), male heads without antennae (MH), pooled thoraces (T), pooled legs (L), female abdomens (FAb), and male abdomens (MAb). In total, 137.99 Gb of clean data were generated, with each sample yielding at least 6.04 Gb. The Q30 percentage exceeded 97.78% across all samples, indicating high sequencing quality.

Clean reads were mapped to the *L. glycinivorella* reference genome, with mapping rates ranging from 66.83% to 80.41%. Based on these alignments, alternative splicing events were predicted, gene models were refined, and novel genes were identified. In total, 5593 novel genes were discovered, of which 1402 received functional annotations (Table 1).

### 3.2. Identification of Chemosensory-Related Genes

Based on transcriptome annotation and homology searches, we identified candidate members of the six major chemosensory gene families, including odorant receptors (ORs), gustatory receptors (GRs), ionotropic receptors (IRs), odorant-binding proteins (OBPs), chemosensory proteins (CSPs), and sensory neuron membrane proteins (SNMPs). Among the soluble olfactory proteins, we identified 52 OBPs and 18 CSPs (Supplementary Tables S3 and S4). All candidate sequences were further validated by ORF prediction. Among them, 44 OBPs and 16 CSPs contained full-length open reading frames. Sequence analysis revealed that most canonical OBPs retain the conserved six-cysteine motif (C-pattern), while CSPs possess four conserved cysteine residues, both of which are important for tertiary structure stability and ligand binding. Both OBP and CSP members were predicted to contain an N-terminal signal peptide and to lack transmembrane domains, consistent with their roles as secreted carrier proteins that bind and transport hydrophobic volatile molecules through the aqueous sensillar lymph.

In addition to soluble proteins, we identified four families of membrane-bound chemosensory genes (Supplementary Tables S5–S8): 76 ORs, of which 54 were full-length; 15 GRs, all full-length; 18 IRs, with 7 full-length; and 4 SNMPs, all full-length. ORs and GRs typically exhibited 6–7 predicted transmembrane domains, consistent with seven-transmembrane chemosensory receptors. SNMPs featured two transmembrane domains and a large extracellular loop, which are hallmark features of CD36-family proteins implicated in olfactory signaling. IRs retained conserved domains and key residues homologous to ionotropic glutamate receptors (iGluRs), reflecting structural conservation. Overall, the structural features of *L. glycinivorella* chemosensory genes closely resemble those reported in other lepidopterans, supporting the reliability of our annotations.

### 3.3. Phylogenetic Analysis of Chemosensory Genes

To infer evolutionary relationships, maximum-likelihood phylogenetic trees were constructed for all six chemosensory gene families (OR, GR, IR, OBP, CSP, and SNMP) using homologs from representative Lepidoptera. In general, *L. glycinivorella* candidates clustered within well-supported, conserved clades of lepidopteran orthologs, providing phylogenetic support for functional inference. Bayesian inference (BI) phylogenetic analyses based on CDS datasets yielded topologies generally consistent with the ML trees, further supporting the robustness of the inferred phylogenetic relationships (Supplementary Figures S1–S6).

**Figure 1:**
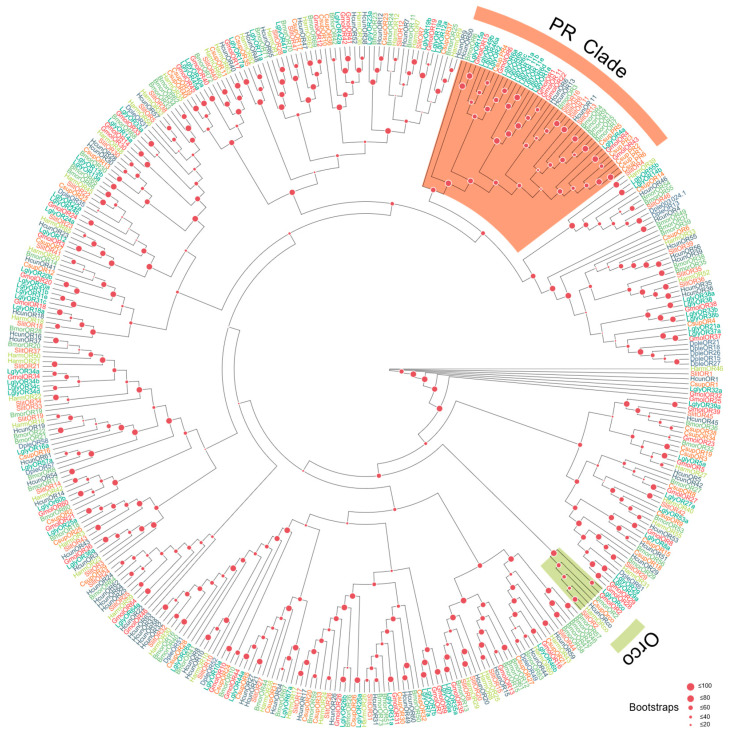
Homology analysis of ORs from *L. glycinivorella* and representative Lepidoptera. The sequences used to construct the phylogenetic tree were derived from the following 7 species: *Grapholita molesta* (Busck, 1916) (Gmol) [54,55]; *Chilo suppressalis* (Csup) [56]; *Helicoverpa armigera* (Harm) [55]; *Bombyx mori* (Linnaeus, 1758) (Bmor); *Danaus plexippus* (Linnaeus, 1758) (Dple); *Hyphantria cunea* (Drury, 1773) (Hcun) [14]; *Spodoptera litura* (Fabricius, 1775) [57]. Different font colors indicate different species, and the shaded colored regions indicate the major clades labeled in the figure.

OR Family (Figure 1): The co-receptor *LglyOrco* formed a distinct, strongly supported clade with Orco orthologs from other lepidopterans, consistent with its conserved role. Excluding Orco, 13 LglyORs grouped within the lepidopteran pheromone receptor (PR) clade, suggesting potential involvement in sex pheromone detection. The remaining LglyORs were distributed across multiple subfamilies: some showed putative one-to-one orthology with reported host-volatile receptors, whereas others formed species-specific clusters, indicating possible functional diversification in host-odor and environmental-cue recognition. The BI tree showed a generally consistent topology with the ML tree, further supporting the phylogenetic placement of LglyOrco and the candidate PR lineage (Supplementary Figure S1).

**Figure 2:**
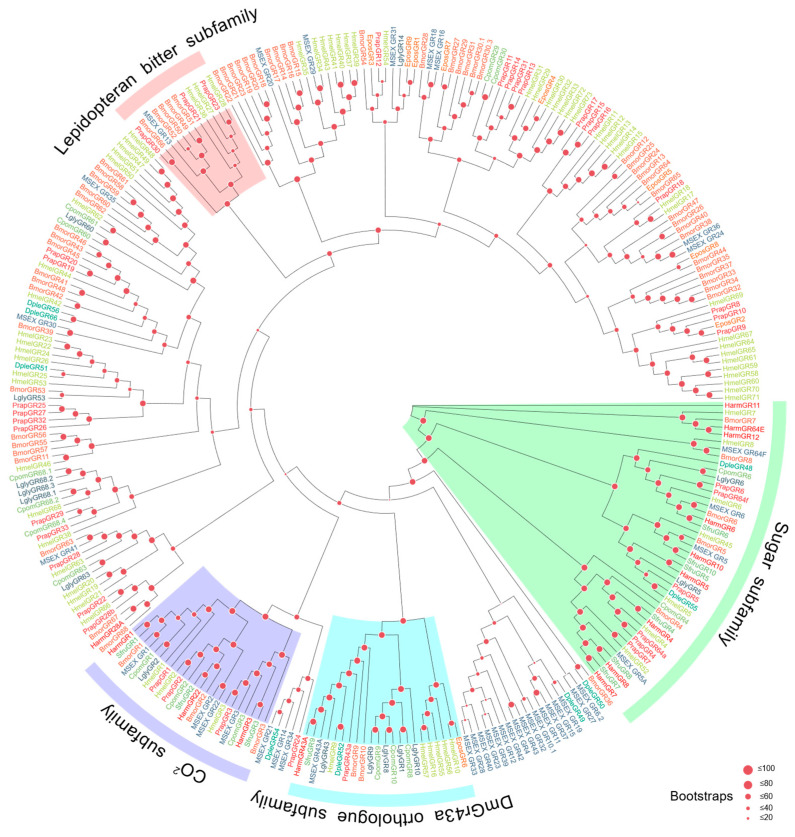
Homology analysis of GRs from *L. glycinivorella* and representative Lepidoptera. The sequences used to construct the phylogenetic tree were derived from the following 9 species: *Cydia pomonella* (Linnaeus, 1758) (Cpom) [58]; *Pieris rapae* (Linnaeus, 1758) (Prap) [59]; *Manduca sexta* (Linnaeus, 1763) (Msex) [60]; *Bombyx mori* (Bmor) [61]; *Heliconius melpomene* (Linnaeus, 1758) (Hmel) [62]; *Helicoverpa armigera* (Harm) [63]; *Spodoptera frugiperda* (J. E. Smith, 1797) (Sfru) [64]; *Danaus plexippus* (Dple) [65]; *Epiphyas postvittana* (Walker, 1863) (Epos) [66]. Different font colors indicate different species, and the shaded colored regions indicate the major clades labeled in the figure.

GR Family (Figure 2): The phylogenetic tree clarified the correspondence between *L. glycinivorella* GR members and major functional subfamilies of lepidopteran GRs. Within the putative CO_2_ receptor clade (GR1/GR2/GR3 lineage), *LglyGR2* clustered with orthologs from other lepidopterans, whereas no GR1- or GR3-like homologs were recovered from our transcriptome dataset. In the sugar receptor-related clade, *LglyGR5* and *LglyGR6* were identified. In addition, no *L. glycinivorella* candidates were assigned to the bitter taste receptor-related clade in the phylogenetic analysis. Bayesian inference analysis recovered a topology largely consistent with the ML tree, supporting the inferred placement of the CO_2_ receptor and sugar receptor-related GR lineages (Supplementary Figure S2).

**Figure 3:**
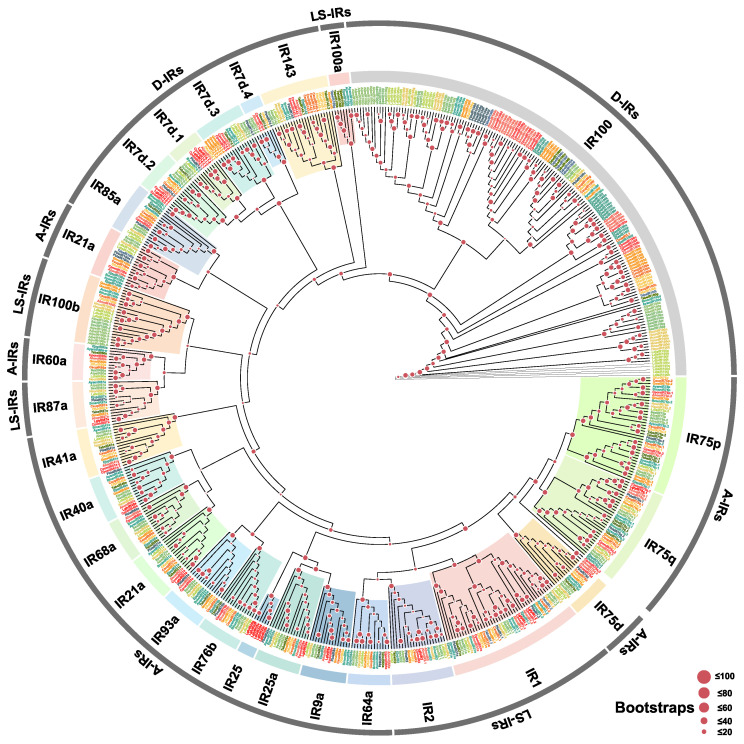
Homology analysis of IRs from *L. glycinivorella* and representative Lepidoptera. The sequences used to construct the phylogenetic tree were derived from the following 14 species: *Cydia pomonella* (Cpom); *Adoxophyes honmai* (Yasuda, 1998) (Ahon); *Papilio bianor* (Cramer, 1777) (Pbia); *Chilo suppressalis* (Csup); *Ostrinia furnacalis* (Guenée, 1854) (Ofur); *Galleria mellonella* (Linnaeus, 1758) (Gmel); *Hyphantria cunea* (Hcun); *Lymantria dispar* (Linnaeus, 1758) (Ldis); *Agrotis ipsilon* (Hufnagel, 1766) (Aips); *Spodoptera exigua* (Hübner, 1808) (Sexi); *Antheraea yamamai* (Guérin-Méneville, 1861) (Ayan); *Bombyx mandarina* (Moore, 1872) (Bman); *Eumeta japonica* (Heylaerts, 1884) (Ejap); *Tuta absoluta* (Meyrick, 1917) (Tabs) [18]. Different font colors indicate different species, and the shaded colored regions indicate the major clades labeled in the figure.

IR family (Figure 3): Among the 18 identified LglyIR genes, phylogenetic analysis classified them into three subfamilies. The Antennal IRs (A-IRs) include two conserved co-receptors, *LglyIR8a* and *LglyIR25a*, as well as a group of tuning receptors—*LglyIR21a*, *LglyIR40a*, *LglyIR75q.1*, and *LglyIR87a*. Additionally, a *L. glycinivorella*-specific clade was identified, which clusters with the co-receptors IR25a and IR8a and comprises four paralogs: *LglyIR25b*, *LglyIR25c*, *LglyIR25d*, and *LglyIR25e*. Within the Lepidoptera-Specific IRs (LS-IRs), three members were identified: *LglyIR1.1*, *LglyIR1.2*, and *LglyIR2*. In the Divergent IRs (D-IRs), only four copies from the IR7d family were detected (*LglyIR7d.1.1*, *LglyIR7d.1.2*, *LglyIR7d.2.1*, and *LglyIR7d.4*). No *L. glycinivorella* homologs were found in the IR100a, IR100b, IR143, or IR85a clades. The BI tree was generally congruent with the ML topology and supported the major IR clades identified in this study (Supplementary Figure S3).

**Figure 4:**
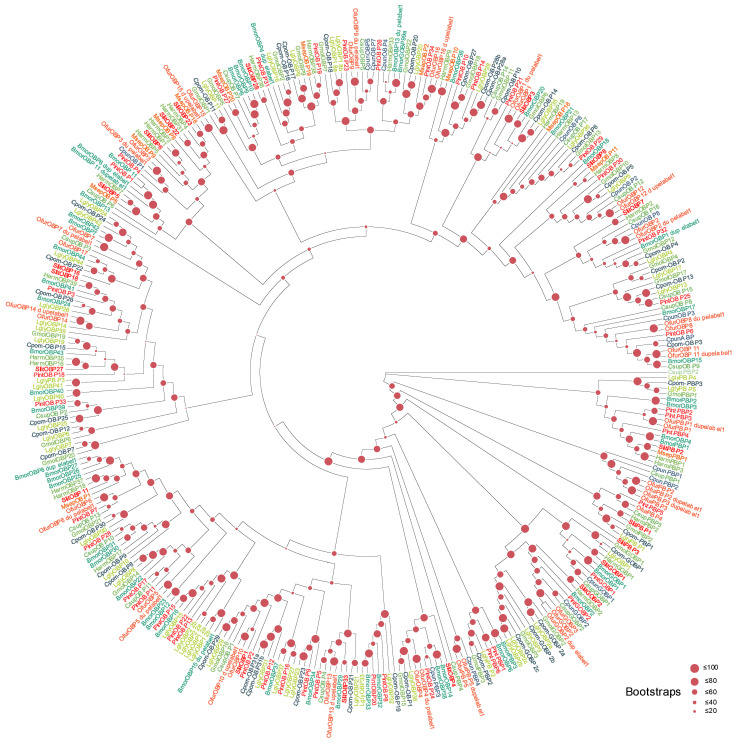
Homology analysis of OBPs from *L. glycinivorella* and other representative insect species. The sequences used to construct the phylogenetic tree were derived from the following 10 species: *Plodia interpunctella* (Hübner, 1813) (Pint); *Ostrinia furnacalis* (Ofur); *Conogethes punctiferalis* (Guenée, 1854) (Cpun); *Chilo suppressalis* (Csup); *Bombyx mori* (Bmor) [67]; *Grapholita molesta* (Gmol); *Spodoptera litura* (Slit); *Helicoverpa armigera* (Harm) [54]; *Cydia pomonella* (Cpom) [68]; *Mythimna separata* (Walker, 1865) (Msep) [69]. Different font colors indicate different species.

OBP Family (Figure 4): Phylogenetic analysis showed that *L. glycinivorella* OBPs are distributed across the major lepidopteran OBP clades. Notably, several LglyOBPs formed sister pairs or small species-specific clades (e.g., *LglyOBP12a/12b* and *LglyOBP18a/18b*), consistent with recent lineage-specific duplications followed by sequence divergence. Bayesian inference analysis yielded a generally consistent topology with the ML tree, further supporting the observed OBP clustering pattern and the inferred lineage-specific duplications (Supplementary Figure S4).

**Figure 5:**
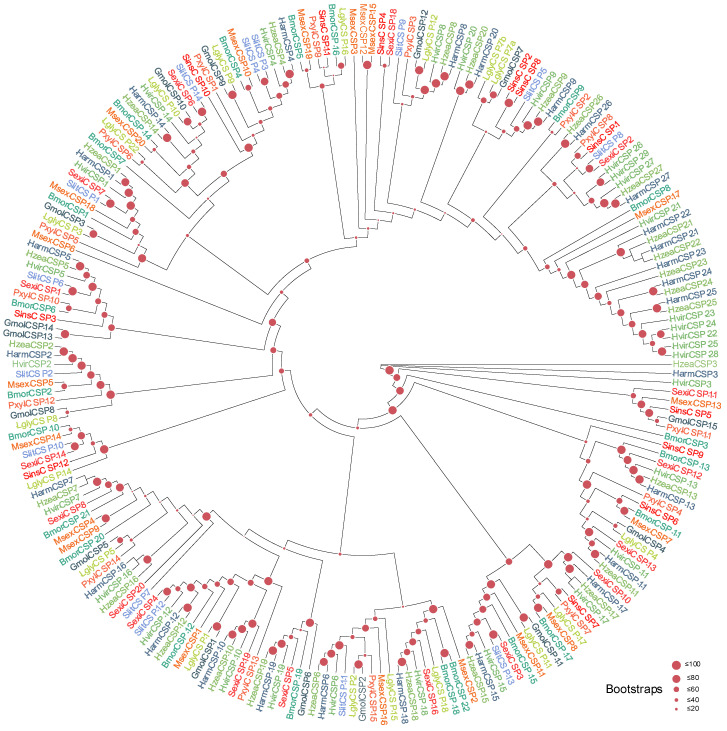
Homology analysis of CSPs from *L. glycinivorella* and other representative insect species. The sequences used to construct the phylogenetic tree were derived from the following 10 species: *Grapholita molesta* (Gmol); *Spodoptera litura* (Slit) [54]; *Streltzoviella insularis* (Staudinger, 1892) (Sins); *Spodoptera exigua* (Sexi) [70]; *Bombyx mori* (Bmor); *Helicoverpa armigera* (Harm); *Heliothis virescens* (Fabricius, 1777) (Hvir); *Plutella xylostella* (Linnaeus, 1767) (Pxyl); *Helicoverpa zea* (Boddie, 1850) (Hzea); *Manduca sexta* (Msex) [71]. Different font colors indicate different species.

CSP family (Figure 5): *L. glycinivorella* CSPs co-clustered with CSPs from representative lepidopteran species across multiple clades, indicating an overall conserved evolutionary pattern. A few members formed closely related pairs or species-specific subclades (e.g., *LglyCSP7a* and *LglyCSP7b*), suggesting possible local gene duplication or expansion. Such lineage-specific changes may contribute to the functional diversification of CSPs beyond olfaction across multiple tissues. The BI tree showed an overall topology consistent with the ML analysis, supporting both the conserved CSP clades and the inferred species-specific expansions (Supplementary Figure S5).

**Figure 6:**
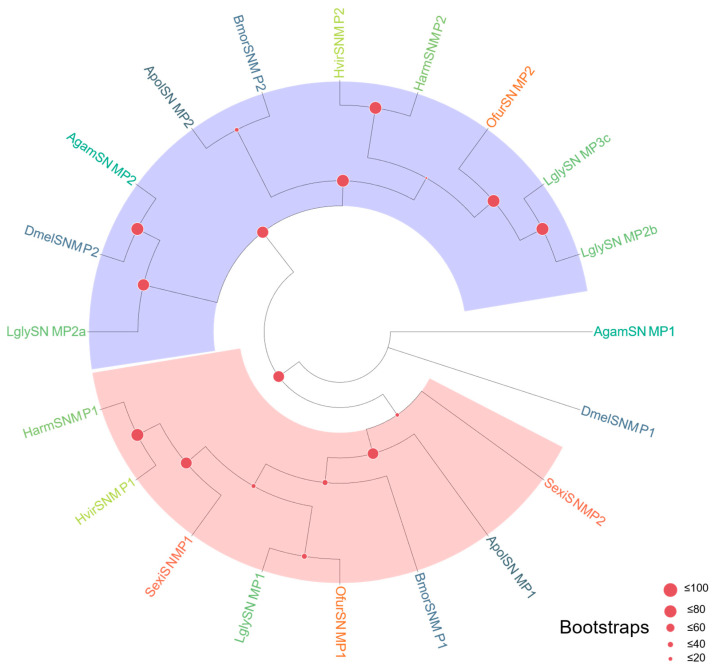
Homology analysis of sensory neuron membrane proteins (SNMPs) from *L. glycinivorella* and other representative insect species. The sequences used to construct the phylogenetic tree were derived from the following 8 species: *Anopheles gambiae* (Giles, 1900) (Agam); *Bombyx mori* (Bmor); *Drosophila melanogaster* (Meigen, 1830) (Dmel); *Helicoverpa armigera* (Harm); *Heliothis virescens* (Hvir); *Ostrinia furnacalis* (Ofur); *Spodoptera exigua* (Sexi); *Antheraea polyphemus* (Cramer, 1775) (Apol) [72]. Different font colors indicate different species, and the shaded colored regions indicate the major clades labeled in the figure.

SNMP Family (Figure 6): SNMP members were clearly divided into two subfamilies, SNMP1 and SNMP2. *LglySNMP1* clustered with SNMP1 homologs from other lepidopterans in a single well-supported clade, consistent with the reported association of SNMP1 with sex pheromone detection. In contrast, *LglySNMP2a*, *LglySNMP2b*, *LglySNMP3c* grouped within the SNMP2 clade, reflecting divergence from SNMP1. Given that SNMP1 has been shown to play an essential role in sex pheromone perception in several lepidopteran species, *LglySNMP1* represents a high-priority candidate for subsequent functional validation. Bayesian inference analysis also supported the separation of SNMP1 and SNMP2 and the phylogenetic placement of LglySNMP1 within the SNMP1 clade (Supplementary Figure S6).

In summary, phylogenetic analyses indicate that the chemosensory-related genes of *L. glycinivorella* exhibit typical lepidopteran evolutionary characteristics and show evidence of potential gene duplication and lineage-specific divergence, providing a foundation for subsequent screening of key functional candidates based on expression profiles, electrophysiological assays, or genetic manipulation. The overall congruence between the ML and BI trees further supports the robustness of these phylogenetic inferences.

### 3.4. Tissue-Specific Expression Analysis of Chemosensory Genes

To characterize the expression distribution of chemosensory-related genes across tissues and sex-biased expression in the antennae of *L. glycinivorella*, we generated clustered heatmaps based on FPKM values for the six gene families (OR, GR, IR, OBP, CSP, and SNMP) and performed differential expression analysis between female and male antennae; significantly differentially expressed genes were visualized in volcano plots.

Overall, the heatmap results showed that, compared with heads (antennae removed), thoraces, legs, and abdomens, genes from the OR, IR, OBP, and SNMP families were generally more highly expressed in antennae, exhibiting a clear antennal-enriched pattern. In contrast, CSP and GR family genes showed broader expression overall, although some members still exhibited relatively higher expression in antennae or displayed sex-biased expression.

At the family level, the OR family was strongly enriched in antennae, and several ORs showed relatively higher expression in female antennae (e.g., *LglyOR13* and *LglyOR15*). The GR family exhibited a relatively dispersed expression pattern but still included sex-differentially expressed genes. The IR family contained only a small number of differentially expressed genes, and no down-regulated genes were detected. Among soluble carrier proteins, the OBP family was primarily antennal-enriched and included sex-differentially expressed genes (e.g., *LglyOBP2* and *LglyOBP8*), whereas the CSP family was broadly expressed, with only a few genes showing sex-biased expression. Finally, the SNMP family showed overall high expression in antennae and a detectable degree of sex-biased expression.

**Figure 7:**
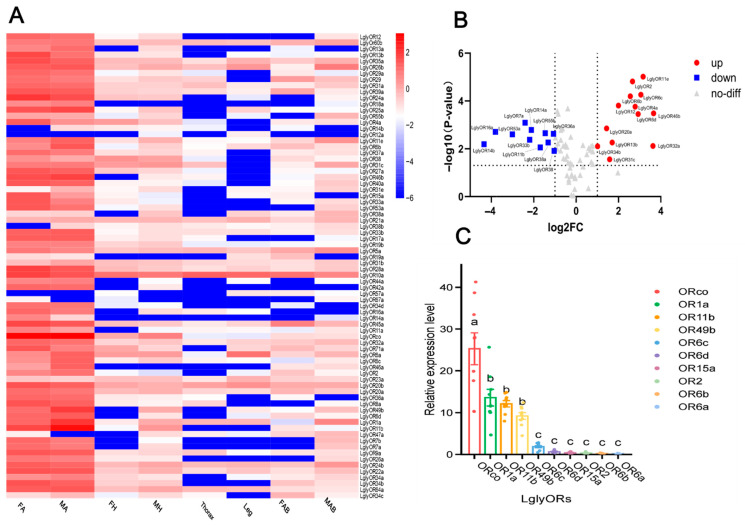
Expression profiles and sex-biased differential expression of OR genes in *L. glycinivorella*, and qRT-PCR assessment of representative candidates. (**A**): Heatmap showing the expression of OR genes in different tissues of *L. glycinivorella*. The color scale ranges from blue (low expression) to red (high expression), representing the normalized expression levels of log10(FPKM). (**B**): Volcano plot of differentially expressed OR genes in *L. glycinivorella*. The horizontal axis represents the log2 fold change in the comparison between female antennae (FA) and male antennae (MA), while the vertical axis shows the −log10(*p*-value). The vertical dashed lines indicate the thresholds of log2FC = −1 and 1, and the horizontal dashed line indicates the significance threshold of *p* = 0.05. Red dots indicate significantly differentially expressed genes (|log2FC| > 1, *p* < 0.05), and gray triangles represent genes without significant differential expression. (**C**): qRT-PCR assessment of ten representative OR genes in male antennae. Relative transcript levels of *LglyORco*, *LglyOR1a*, *LglyOR11b*, *LglyOR49b*, *LglyOR6c*, *LglyOR6d*, *LglyOR15a*, *LglyOR2*, *LglyOR6b*, and *LglyOR6a* were quantified using the 2^−ΔΔCt^ method with actin as the reference gene. Bars represent mean ± SEM, and dots indicate individual qPCR wells (technical replicates, *n* = 9). Different letters denote significant differences among genes (one-way ANOVA with Tukey’s multiple comparisons, *p* < 0.05; based on technical replicates).

Multiple OR genes in *L. glycinivorella* were significantly enriched in male antennae, whereas several genes, including *LglyOR13*, *LglyOR15*, *LglyOR32a*, and *LglyOR35a*, exhibited tissue-specific high expression in female antennae, heads, or abdomens (Figure 7A). The differential-expression volcano plot (Figure 7B) further indicated sex-biased expression between female and male antennae: 12 OR genes were significantly higher in male antennae (e.g., *LglyOR11e*, *LglyOR2*, *LglyOR6c*, and *LglyOR4a*), whereas five genes (e.g., *LglyOR10*, *LglyOR32a*, and *LglyOR35a*) showed significantly lower expression in female antennae; most remaining ORs did not exhibit significant changes.

To strengthen the robustness of transcriptome-based expression profiling, we performed qRT-PCR for ten representative OR genes using an independent batch of male antennae (Figure 7C). The qRT-PCR results revealed a clear expression hierarchy among these candidates in male antennae, providing independent experimental support for the transcriptome-derived OR expression profile. ORs were prioritized for validation because OR-mediated olfaction constitutes a core molecular basis of odor detection in insects and is directly relevant to antennal odorant recognition.

**Figure 8:**
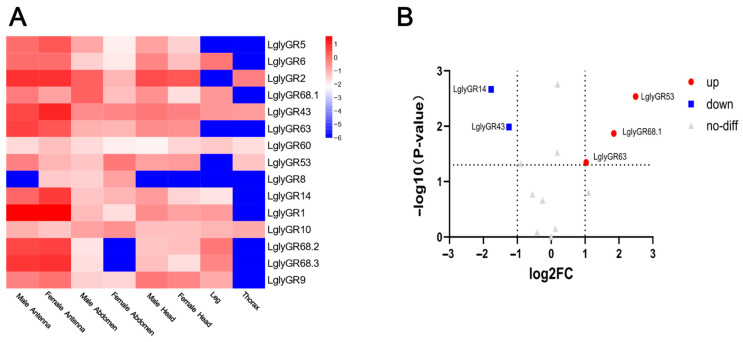
Tissue expression profiles and differential expression of gustatory receptor (GR) genes in *L. glycinivorella*. (**A**): Heatmap showing the expression of GR genes in different tissues of *L. glycinivorella*. The color scale ranges from blue (low expression) to red (high expression), representing the normalized expression levels of log10(FPKM). (**B**): Volcano plot of differentially expressed GR genes in *L. glycinivorella*. The horizontal axis represents the log2 fold change in the comparison between female antennae (FA) and male antennae (MA), while the vertical axis shows the −log10(*p*-value). The vertical dashed lines indicate the thresholds of log2FC = −1 and 1, and the horizontal dashed line indicates the significance threshold of *p* = 0.05. Red dots indicate significantly differentially expressed genes (|log2FC| > 1, *p* < 0.05), and gray triangles represent genes without significant differential expression.

Most LglyGR genes showed higher expression in the antennae, particularly in male antennae, such as *LglyGR5*, *LglyGR6*, *LglyGR2*, and *LglyGR43*. In contrast, *LglyGR68.1*, *LglyGR63*, *LglyGR60*, *LglyGR53*, *LglyGR8*, *LglyGR14*, *LglyGR10*, *LglyGR68.2*, *LglyGR68.3*, and *LglyGR9* exhibit higher expression in thorax, legs, or female tissues (Figure 8A). Some of these genes are expressed more highly in female antennae or abdomen, indicated in blue, suggesting they may participate in gender-specific or non-olfactory-related sensory functions. Further analysis using a volcano plot examined the differential expression of *L. glycinivorella* GR genes between male and female antennae (Figure 8B). It was found that *LglyGR53* and *LglyGR68.1* were significantly upregulated in male antennae, while *LglyGR14* and *LglyGR43* were significantly downregulated in female antennae. The remaining genes did not show significant differential expression.

**Figure 9:**
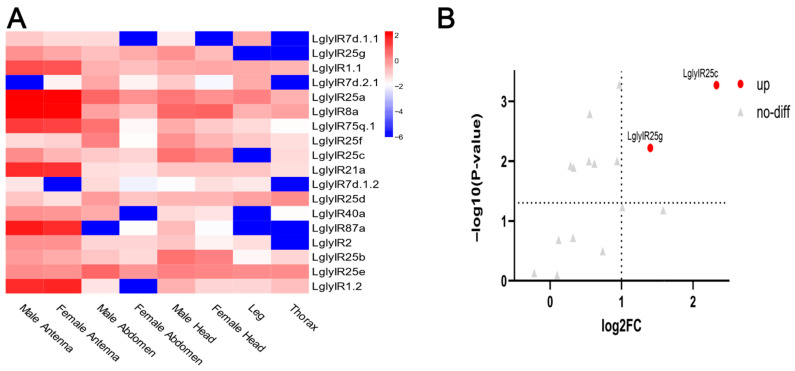
Tissue expression profiles and differential expression of ionotropic receptor (IR) genes in *L. glycinivorella*. (**A**): Heatmap showing the expression of IR genes in different tissues of *L. glycinivorella*. The color scale ranges from blue (low expression) to red (high expression), representing the normalized expression levels of log_10_(FPKM). (**B**): Volcano plot of differentially expressed IR genes in *L. glycinivorella*. The horizontal axis represents the log2 fold change in the comparison between female antennae (FA) and male antennae (MA), while the vertical axis shows the −log10(*p*-value). The vertical dashed lines indicate the thresholds of log2FC = 1, and the horizontal dashed line indicates the significance threshold of *p* = 0.05. Red dots indicate significantly differentially expressed genes (|log2FC| > 1, *p* < 0.05), and gray triangles represent genes without significant differential expression.

Among the antennal IRs (A-IRs), the co-receptor *LglyIR8a* was highly expressed in both male and female antennae and showed detectable expression in heads, abdomens, and legs, whereas *LglyIR25a* exhibited extremely low expression (Figure 9A,B). Its paralogs *LglyIR25b*–e displayed divergent expression patterns: *LglyIR25c* was highly expressed in male antennae; *LglyIR25b* was enriched in thoraces and legs; LglyIR25d was primarily expressed in male abdomens; and *LglyIR25e* showed the highest expression in legs. Among the tuning receptors, *LglyIR75q*.1 showed the highest expression in male antennae, and LglyIR40a was also enriched in male antennae, whereas *LglyIR21a* and *LglyIR87a* were expressed at low levels in antennae; Within the lepidopteran-specific IRs (LS-IRs), *LglyIR2* showed the highest expression in male antennae, followed by *LglyIR1.2*, while *LglyIR1*.1 was expressed at a low level in male antennae; all three showed very low or undetectable expression in other tissues. In the divergent IRs (D-IRs), *LglyIR7d.2.1* displayed the highest expression in male antennae, and *LglyIR7d.1.1*, *LglyIR7d.1.2*, and *LglyIR7d.4* also showed male antennae as their primary expression site, with minimal or no expression in other tissues. Consistent with the phylogenetic analysis, no IR100a-, IR100b-, IR143-, or IR85a-like homologs were recovered from our transcriptome dataset, and no corresponding expression signals were observed in the heatmap. Differential expression analysis between female and male antennae further indicated that *LglyIR25b* and *LglyIR25d* were significantly upregulated in male antennae, with *LglyIR25b* showing the strongest statistical support; no other IR genes showed significant sex-biased expression in antennae.

**Figure 10:**
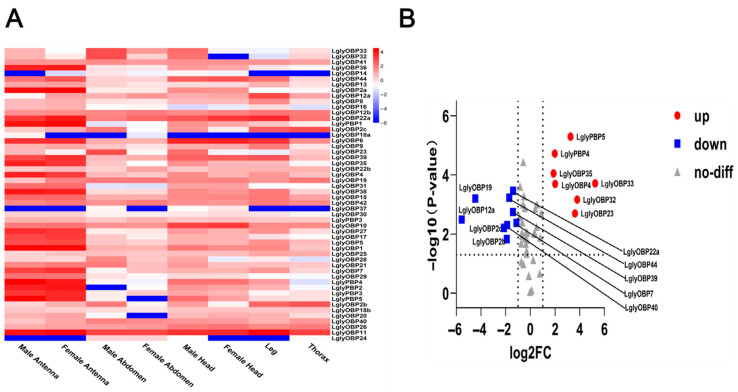
Tissue expression profiles and differential expression of odorant-binding protein (OBP) genes in *L. glycinivorella*. (**A**): Heatmap showing the expression of OBP genes in different tissues of *L. glycinivorella*. The color scale ranges from blue (low expression) to red (high expression), representing the normalized expression levels of log_10_(FPKM). (**B**): Volcano plot of differentially expressed OBP genes in *L. glycinivorella*. The horizontal axis represents the log2 fold change in the comparison between female antennae (FA) and male antennae (MA), while the vertical axis shows the −log10(*p*-value). The vertical dashed lines indicate the thresholds of log2FC = −1 and 1, and the horizontal dashed line indicates the significance threshold of *p* = 0.05. Red dots indicate significantly differentially expressed genes (|log2FC| > 1, *p* < 0.05), and gray triangles represent genes without significant differential expression.

The heatmap showed that most LglyOBP genes were highly expressed in antennae, with pronounced enrichment in both male and female antennae (Figure 10A). Notably, several OBPs (e.g., *LglyOBP33*, *LglyOBP4*, and *LglyOBP23*) exhibited higher expression in male antennae. Volcano-plot analysis further indicated that multiple OBP genes were significantly differentially expressed between antennae and non-antennal tissues, with *LglyOBP33*, *LglyOBP4*, and *LglyOBP23* significantly upregulated and *LglyOBP19* and *LglyOBP12a* significantly downregulated (Figure 10B).

**Figure 11:**
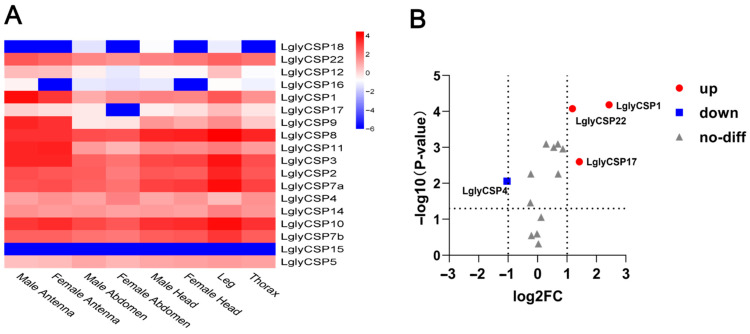
Tissue expression profiles and differential expression of chemosensory protein (CSP) genes in *L. glycinivorella*. (**A**): Heatmap showing the expression of CSP genes in different tissues of *L. glycinivorella*. The color scale ranges from blue (low expression) to red (high expression), representing the normalized expression levels of log10(FPKM). (**B**): Volcano plot of differentially expressed CSP genes in *L. glycinivorella*. The horizontal axis represents the log2 fold change in the comparison between female antennae (FA) and male antennae (MA), while the vertical axis shows the −log10(*p*-value). The vertical dashed lines indicate the thresholds of log2FC = −1 and 1, and the horizontal dashed line indicates the significance threshold of *p* = 0.05. Red dots indicate significantly differentially expressed genes (|log2FC| > 1, *p* < 0.05), and gray triangles represent genes without significant differential expression.

The heatmap showed that most LglyCSP genes were highly expressed in the antennae of *L. glycinivorella*, with clear sex-biased patterns. *LglyCSP1*, *LglyCSP2*, *LglyCSP8*, *LglyCSP11*, and *LglyCSP14* exhibited higher expression in male antennae, whereas *LglyCSP15* and *LglyCSP17* showed higher expression in female antennae (Figure 11A). In addition, some genes (e.g., *LglyCSP2* and *LglyCSP3*) displayed relatively high expression across multiple tissues. Volcano-plot analysis further indicated that *LglyCSP1* and *LglyCSP22* were significantly upregulated in male antennae, whereas *LglyCSP17* was significantly upregulated in female antennae (Figure 11B).

**Figure 12:**
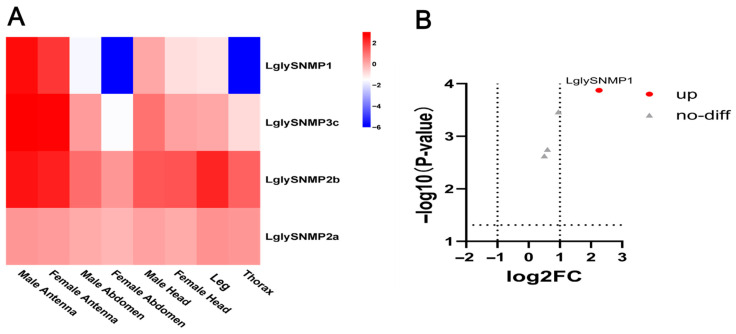
Tissue expression profiles and differential expression of sensory neuron membrane protein (SNMP) genes in *L. glycinivorella*. (**A**): Heatmap showing the expression of SNMP genes in different tissues of *L. glycinivorella*. The color scale ranges from blue (low expression) to red (high expression), representing the normalized expression levels of log10(FPKM). (**B**): Volcano plot of differentially expressed SNMP genes in *L. glycinivorella*. The horizontal axis represents the log2 fold change in the comparison between female antennae (FA) and male antennae (MA), while the vertical axis shows the −log10(*p*-value). The vertical dashed lines indicate the thresholds of log2FC = −1 and 1, and the horizontal dashed line indicates the significance threshold of *p* = 0.05.Red dots indicate significantly differentially expressed genes (|log2FC| > 1, *p* < 0.05), and gray triangles represent genes without significant differential expression.

*LglySNMP1* exhibited significantly high expression in both male and female antennae, while its expression was markedly lower in other tissues such as the abdomen, head, and thorax. *LglySNMP2a* and *LglySNMP2b* also showed relatively high expression in antennae and head, but their expression was broader compared to *LglySNMP1*, extending to legs and thorax (Figure 12A). The expression pattern of *LglySNMP3c* resembled that of *LglySNMP1*, though it was nearly absent in the abdomen and thorax. Volcano plot analysis of specific comparison groups (e.g., antennae vs. other tissues) further revealed that only *LglySNMP1* was significantly upregulated in this contrast, whereas the other three genes did not show significant differential expression (Figure 12B).

## 4. Discussion

A thorough dissection of the chemosensory system in *L. glycinivorella* provides molecular targets and a theoretical basis for behavior-based green pest management strategies within integrated pest management (IPM), such as pheromone trapping and the screening of host-volatile attractants or repellents. Chemosensory molecules—including ORs, GRs, IRs, and peripheral auxiliary proteins such as OBPs—represent key entry points for “reverse chemical ecology” approaches aimed at discovering semiochemicals and optimizing behavioral control tactics [73]. However, the molecular mechanisms underlying chemical perception in *L. glycinivorella* remain poorly understood. Here, we systematically identified chemosensory-related genes through multi-tissue transcriptome sequencing and bioinformatic analyses and characterized their sequence features, phylogenetic relationships, and tissue-specific expression patterns, thereby providing a resource for subsequent functional validation and ligand screening of candidate genes [74].

ORs are core receptors in insect olfaction and typically form heteromeric complexes with the conserved co-receptor ORco to mediate signal transduction. In this study, we identified LglyORco, providing a basis for subsequent heterologous expression and ligand screening. In the silkworm (*Bombyx mori*), loss of ORco significantly reduces olfactory sensitivity and impairs related behaviors, underscoring the functional conservation and essentiality of Orco [75]. In Lepidoptera, pheromone receptors (PRs) represent a specialized clade within the OR family and mediate mate recognition. For instance, *BmOR1* in *B. mori* has been functionally validated to detect a sex pheromone component [76], and PR lineages have undergone species-specific expansion and functional diversification across moth species [77]. Here, we identified 13 candidate PRs in *L. glycinivorella*, indicating a potentially diverse receptor repertoire for sex pheromone detection; however, functional assignment will require validation using sex- and tissue-specific expression patterns and ligand-response profiles from heterologous expression assays. A limitation of this study is the lack of paired qRT-PCR validation for FA–MA DEGs and the absence of functional assays for candidate ORs. Therefore, the PR candidates and female-biased ORs proposed here should be regarded as hypothesis-driven priorities for subsequent experimental validation. In contrast, several ORs in our dataset (e.g., *LglyOR13* and *LglyOR15*) were upregulated in female antennae, more plausibly indicating roles in host location or oviposition-site selection. Notably, phylogenetic placement alone does not strictly predict ligand type: for example, *CpomOR3* in codling moth (*Cydia pomonella*), initially annotated as PR-like, was shown to respond to the host volatile pear ester [78]; similarly, *HassOR31* in *Helicoverpa assulta* (Guenée, 1852) is highly expressed in the ovipositor and is implicated in host-volatile detection [79]. Therefore, as working hypotheses, candidate ORs can be prioritized into two functional categories: (1) PR candidates likely associated with sex pheromone communication, and (2) female-biased ORs potentially involved in recognizing host- or oviposition-related chemical cues. This framework provides biologically plausible priorities for future functional validation.

GRs primarily mediate insect perception of contact chemical stimuli—such as sugars and bitter compounds—as well as gaseous cues such as CO_2_, providing a molecular basis for feeding selection, host recognition, and oviposition decisions [63]. In this study, we identified 15 candidate LglyGR genes from multi-tissue transcriptomes, including putative GR43a-like (“fructose receptor”) members, candidate CO_2_ receptor-related genes, and sugar receptor-related members, suggesting that *L. glycinivorella* retains molecular components for detecting nutritional signals and CO_2_-related cues [63]. Functional evidence from other insects supports these annotations. In Drosophila, Gr43a has been confirmed as a highly specific fructose receptor and internal nutrient sensor that modulates feeding in response to physiological fructose levels [80]. Similar functions have been reported in Lepidoptera: *Helicoverpa armigera HaGr4* responds specifically to fructose [81], while *HaGr9* (reported as identical to or closely related to *HaGr4*) also responds to D-maltose and D-galactose in addition to fructose [82]. Likewise, *Ostrinia furnacalis* OfurGr43 has been shown to detect both D-fructose and D-sorbitol [61]. In *L. glycinivorella*, *LglyGR1*, *LglyGR8*, *LglyGR9*, *LglyGR10*, and *LglyGR43* were assigned to the GR43a-related clade, suggesting that this pest may exhibit sensitivity to fructose-associated cues.

Notably, we did not recover homologs assigned to the canonical bitter GR lineage from our dataset [83], which may reflect its specialized oligophagous lifestyle. Regarding CO_2_ perception, lepidopterans typically retain a conserved CO_2_ receptor lineage composed of three subunits (GR1, GR2, and GR3), which are predominantly expressed in labial palp sensilla and mediate behavioral responses to CO_2_ [63]. For example, in *H. armigera*, co-expression of *HarmGr1* with HarmGr3 (or *HarmGr2*) in heterologous systems elicits robust electrophysiological responses to bicarbonate/CO_2_, and all three receptors are co-expressed in the same labial palp neuron, supporting a “GR complex-mediated CO_2_ detection” model [84]. Similarly, in the fall webworm *Hyphantria cunea*, a binary system expressing *HcunGR1* and *HcunGR3* produces concentration-dependent responses to high CO_2_, while a ternary combination (*HcunGR1/2/3*) is activated by CO_2_-containing solutions [85]. In *L. glycinivorella*, however, we identified only *LglyGR2* and did not recover GR1- or GR3-like homologs. This pattern may reflect one or more of the following: (1) labial palps were not separately sequenced, resulting in failure to recover low-abundance GR1/GR3 transcripts; (2) CO_2_ detection may rely on GR2 alone or GR2 in combination with non-canonical subunits; or (3) CO_2_-sensing capability may have undergone functional reduction. Accordingly, *LglyGR2* should be interpreted cautiously as a potential component of a CO_2_-sensing module. Future work should prioritize verifying its expression enrichment in key organs such as labial palps and conducting functional assays using CO_2_ or bicarbonate as primary candidate stimuli [84].

In this study, phylogenetic analysis of the 18 identified LglyIR genes in *L. glycinivorella* assigned them to the three IR subfamilies defined by Yin et al. (2021): antennal IRs (A-IRs), lepidopteran-specific IRs (LS-IRs), and divergent IRs (D-IRs) [18]. Within the A-IRs, *LglyIR8a* and *LglyIR25a* clustered with conserved co-receptors, whereas *LglyIR21a*, *LglyIR40a*, *LglyIR75q.1*, and *LglyIR87a* fell into their respective tuning receptor clades [25]. Notably, we recovered a *L. glycinivorella*-specific clade comprising *LglyIR25b*–e that grouped with IR25a, suggesting lineage-specific duplication of IR25 in this species. Expression analysis further indicated divergence within this clade: *LglyIR25c* was highly expressed in male antennae; *LglyIR25b* was enriched in thoraces and legs; *LglyIR25d* was primarily expressed in male abdomens; and *LglyIR25e* showed the highest expression in legs. Differential expression analysis further showed that *LglyIR25b* and *LglyIR25d* were significantly upregulated in male antennae, suggesting potential roles in male-biased chemosensory processes. Several tuning receptors exhibited high expression in male antennae, including *LglyIR75q.1*, *LglyIR2*, and *LglyIR40a*, suggesting potential involvement in male-biased behaviors such as mate seeking and host-related odor detection. Within the LS-IRs, in addition to LglyIR2, both *LglyIR1.1* and *LglyIR1.2* were predominantly expressed in male antennae and showed minimal or no expression in other tissues, consistent with lepidopteran-specific olfactory tuning receptors. In the D-IRs, only four members of the IR7d family (*LglyIR7d.1.1*, *LglyIR7d.1.2*, *LglyIR7d.2.1*, and *LglyIR7d.4*) were recovered, and all were primarily expressed in male antennae. Typical D-IR lineages such as IR100a, IR100b, IR143, and IR85a were not recovered from our dataset, and no corresponding expression signals were observed in the heatmap. Given that D-IRs in insects such as Drosophila are mainly implicated in gustation and perception of non-volatile compounds [28,29,30], this pattern may be associated with extreme host specialization in *L. glycinivorella*. Functionally, IRs often operate as heteromeric complexes composed of a co-receptor and a tuning receptor to detect acidic volatiles. For example, the Drosophila IR64a/IR8a complex responds to acidic odors [86]; in mosquitoes, IR8a-mediated pathways contribute to the detection of human-derived acidic compounds such as lactic acid [87]; and in *H. armigera*, IR8a is required for acetic acid attraction [88]. In summary, although the IR repertoire of *L. glycinivorella* is smaller than that of many other lepidopterans [18], its members exhibit pronounced tissue specificity and sex-biased expression, particularly the enrichment of multiple tuning receptors in male antennae, while retaining candidates potentially involved in acid and microenvironmental sensing. These features may reflect adaptation to the chemosensory ecology of its soybean host.

We identified 52 LglyOBP genes, including canonical forms and atypical subtypes (Minus-C, Dimer-C). The reduced cysteine motifs in Minus-C OBPs likely alter ligand-binding pocket flexibility, facilitating functional diversification [89,90]. Expression profiling revealed that *LglyOBP2* and *LglyOBP8* are antenna-enriched, with *LglyOBP8* showing significant female bias. This pattern suggests a specific role in detecting host volatiles or oviposition cues, consistent with female-biased OBPs in other lepidopterans [91]. However, as expression patterns are correlative, future ligand-binding assays and behavioral tests are required to validate their specific ligand spectra and rule out functional redundancy [92].

We observed broad tissue distribution for LglyCSP members, supporting their dual roles in both chemosensation and non-olfactory physiological processes (e.g., development, immunity, and xenobiotic response) [93]. While some CSPs in other species mediate olfactory signaling or dietary adaptation [94,95], the ubiquitous expression of LglyCSPs implies a broader functional divergence beyond odorant transport. Additionally, although CSPs have been linked to insecticide resistance in other insects [96], their specific contributions to *L. glycinivorella*’s environmental adaptation and stress response remain to be elucidated through direct functional validation.

SNMPs belong to the CD36-related family of transmembrane proteins and are typically classified into two subfamilies in the insect peripheral olfactory system: SNMP1 and SNMP2 [35]. In this study, we identified one *LglySNMP1* and three *LglySNMP2* genes in *L. glycinivorella.* Phylogenetic analysis showed that *LglySNMP1* clustered with lepidopteran SNMP1 homologs that have been implicated in sex pheromone detection. Consistently, *LglySNMP1* showed higher expression in male antennae, in line with the reported role of SNMP1 in pheromone-sensitive olfactory neurons and pheromone signal transduction [35,72]. For example, in *Helicoverpa armigera*, CRISPR-mediated knockout of SNMP1 markedly reduced male electrophysiological responses to long-chain sex pheromone components and impaired upwind flight orientation and behavioral responses to calling females, supporting an essential role of SNMP1 in sex pheromone detection [72]. Therefore, *LglySNMP1* represents a high-priority candidate for functional validation of sex pheromone signaling in *L. glycinivorella*.

In contrast, the three *LglySNMP2* genes identified here exhibited broader expression across multiple tissues, suggesting that their functions may extend beyond direct involvement in sex pheromone recognition [72]. In various moth species, SNMP2 is mainly localized in support cells of olfactory sensilla and has been proposed to participate in transport or clearance of lipophilic compounds, including pheromone degradation products, from the sensillar lymph, thereby contributing to microenvironmental homeostasis and signal termination. Experimental evidence from *Heliothis virescens* and *Bombyx mori* supports a role of SNMP2 in uptake of long-chain fatty acids (pheromone catabolites) and sensillar “lymph clearance” processes [97,98]. Accordingly, *LglySNMP2* members are more likely to function in supportive roles within the peripheral olfactory system, such as maintaining sensillar microenvironment stability and mediating clearance mechanisms. Future studies could combine cellular localization (e.g., in situ hybridization), RNAi or CRISPR-based knockdown/knockout, and integrated electrophysiological and behavioral assays to further dissect the distinct roles of *LglySNMP1* and *LglySNMP2* [72,97].

## 5. Conclusions

In summary, this study systematically identified the chemosensory gene repertoire of *L. glycinivorella* based on multi-tissue transcriptome sequencing, including 76 ORs, 15 GRs, 18 IRs, 52 OBPs, 18 CSPs, and 4 SNMPs. We comprehensively characterized their sequence features, phylogenetic relationships, and tissue-specific expression profiles. The results indicate that, while retaining core olfactory and gustatory pathways, this monophagous pest exhibits marked functional specialization and contraction of several chemosensory gene families relative to polyphagous lepidopterans, and multiple canonical lineages—such as bitter GRs and several divergent IR lineages (e.g., IR100/IR85a)—were not recovered from our dataset. This streamlining may reflect adaptation to the chemical ecology of its sole host plant, soybean. Concurrently, key receptors and auxiliary proteins displayed pronounced differentiation across sexes and tissues: male antennae were enriched for numerous ORs, IRs, and *LglySNMP1*, suggesting important roles in sex pheromone communication and host-volatile perception; in contrast, female-biased or abdomen-enriched expression of genes such as *LglyOBP2*, *LglyOBP8*, *LglyOR13* and *LglyOR15* points to potential involvement in host location and oviposition decisions. This study further highlights functionally informative candidates: male-biased *LglySNMP1* and candidate PRs represent priorities for validation in pheromone- or host volatile-based behavioral manipulation strategies, whereas female- or abdomen-enriched genes offer entry points for disrupting oviposition behavior. In addition, the presence of a GR43a-related clade supports the capacity for detecting nutritional cues such as fructose, whereas the incomplete CO_2_ receptor module in our dataset (only *LglyGR2* recovered) suggests potential divergence in CO_2_-related sensing. Overall, delineating the composition and expression landscape of chemosensory genes in *L. glycinivorella* provides genetic resources and a conceptual basis for green pest control strategies grounded in “reverse chemical ecology.” Future efforts should prioritize ligand deorphanization, neural localization, and behavioral validation to translate these molecular candidates into field applications.

## Figures and Tables

**Table 1:** Summary statistics of sequencing data.

Samples	Clean Reads	Clean Bases	GC Content	≥Q30 (%)
FA	21,027,433	6,296,277,380	0.425733333	0.9796
FAB	20,524,178.67	6,139,020,059	0.439433333	0.984133333
H	21,191,536	6,343,141,690	0.443166667	0.984033333
Leg	21,073,984.67	6,305,114,227	0.419933333	0.9828
MA	21,036,382	6,293,557,836	0.4264	0.9827
MAb	20,726,886.67	6,192,582,732	0.457666667	0.984266667
MH	21,337,162.67	6,384,668,444	0.448233333	0.983366667
T	20,846,713.67	6,237,254,883	0.416766667	0.983566667

(1) Samples: Sample analysis ID; (2) Clean reads: Total number of paired-end reads in clean data; (3) Clean bases: Total number of bases in clean data; (4) GC content: GC percentage in clean data, i.e., the proportion of guanine (G) and cytosine (C) bases among all bases in the clean data; (5) Q30%: Percentage of bases in clean data with a quality score greater than or equal to 30.

## Data Availability

The raw transcriptome sequencing data supporting this study have been deposited in the NCBI Sequence Read Archive (SRA) under BioProject accession number PRJNA1434332. The corresponding BioSample and SRA run accession numbers are provided in Supplementary Table S1. Additional data supporting the findings of this study are available within the article and its Supplementary Materials.

## Supplementary Materials

The following supporting information can be downloaded at: https://www.mdpi.com/article/10.3390/biology15060505/s1, Table S1: BioSample and SRA accession numbers for transcriptome samples of *L. glycinivorella*. Table S2: Primers used for qRT-PCR analysis of selected odorant receptor genes in *L. glycinivorella*. Table S3: List of candidate CSP genes in *L. glycinivorella*. Table S4: List of candidate OBP genes in *L. glycinivorella*. Table S5: List of candidate OR genes in *L. glycinivorella*. Table S6: List of candidate GR genes in *L. glycinivorella*. Table S7: List of candidate IR genes in *L. glycinivorella*. Table S8: List of candidate SNMP genes in *L. glycinivorella*. Figure S1. BI phylogenetic tree of ORs from *L. glycinivorella* and representative lepidopteran species based on CDS sequences. Figure S2. BI phylogenetic tree of GRs from *L. glycinivorella* and representative lepidopteran species based on CDS sequences. Figure S3. BI phylogenetic tree of IRs from *L. glycinivorella* and representative lepidopteran species based on CDS sequences. Figure S4. BI phylogenetic tree of OBPs from *L. glycinivorella* and representative lepidopteran species based on CDS sequences. Figure S5. BI phylogenetic tree of CSPs from *L. glycinivorell* and representative lepidopteran species based on CDS sequences. Figure S6. BI phylogenetic tree of SNMPs from *L. glycinivorell* and representative lepidopteran species based on CDS sequences.

## References

[1] Yang M., Wang Y., Dai P., Feng D., Hughes A.C., Li H., Zhang A. (2025). Sympatric diversity pattern driven by the secondary contact of two deeply divergent lineages of the soybean pod borer *Leguminivora glycinivorella*. Integr. Zool..

[2] Cui J., Qiao F., Qin B., Xu J., Zhao L., Shi S. (2023). Oviposition selectivity and larval fitness of soybean pod borer, *Leguminivora glycinivorella* (Lepidoptera: Olethreutidae), to different soybean varieties. Chin. J. Oil Crop Sci..

[3] Hu D., Yang X., Feng J., Zhang X. (2014). Advances in the research and application of sex pheromone of soybean pod borer, *Leguminivora glycinivorella* (Matsumura). Chin. J. Pestic. Sci..

[4] Yang M., Wang Z., Wang R., Zhang X., Li M., Xin J., Qin Y., Zhang C., Meng F. (2020). Transcriptomic and proteomic analyses of the mechanisms of overwintering diapause in soybean pod borer (*Leguminivora glycinivorella*). Pest Manag. Sci..

[5] Fang Q., Cao Y., Oo T.H., Zhang C., Yang M., Tang Y., Wang M., Zhang W., Zhang L., Zheng Y. (2024). Overexpression of cry1c enhances resistance against soybean pod borer (*Leguminivora glycinivorella*) in soybean. Plants.

[6] Fei H., Cui J., Zhu S., Xia Y., Xing Y., Gao Y., Shi S. (2024). Integrative analyses of transcriptomics and metabolomics in immune response of *Leguminivora glycinivorella* Mats to *Beauveria bassiana* infection. Insects.

[7] Xue J.-Z., Tariq T., Shen Z., Zhang Y.-H., Tang L.-D., Luo R.-B., Sun Y., Hu C.-C., Zang L.-S. (2025). Eri silkworm eggs as a superior factitious host for mass rearing *Trichogramma leucaniae*, the key natural enemy of soybean pod borer. Biol. Control.

[8] Yoshimura H., Tabuchi K., Konishi K. (2023). Ecological aspect of the larval parasitoid *Diadegma hiraii* (Hymenoptera: Ichneumonidae) as a potential biological control agent of soybean pod borer *Leguminivora glycinivorella* (Lepidoptera: Tortricidae). Environ. Entomol..

[9] Pei T., Cui X., Shi S., Gao Y. (2024). An introductory review on the common brown leafhopper (*Orosius orientalis*): A new soybean pest. Oil Crop Sci..

[10] Leal W.S. (2026). Odorant reception in insects: Functional and evolutionary perspectives. Annu. Rev. Entomol..

[11] Wang B., Jacquin-Joly E., Wang G. (2025). The role of (E)-β-farnesene in tritrophic interactions: Biosynthesis, chemoreception, and evolution. Annu. Rev. Entomol..

[12] Agnihotri A.R., Roy A.A., Joshi R.S. (2016). Gustatory receptors in Lepidoptera: Chemosensation and beyond. Insect Mol. Biol..

[13] Wicher D., Miazzi F. (2021). Functional properties of insect olfactory receptors: Ionotropic receptors and odorant receptors. Cell Tissue Res..

[14] Wang Y., Qu X., Tian Z., Zhou H., Yu Z., Zhou Y., Ren B. (2024). Molecular basis of camphor repellency in *Hyphantria cunea*. Pestic. Biochem. Physiol..

[15] Wang Y., Qiu L., Wang B., Guan Z., Tan Y., Zhao Q., Xu P., Guo H., Jin Y., Zhang J. (2024). Structural basis for odorant recognition of the insect odorant receptor OR-Orco heterocomplex. Science.

[16] Zhao J., Chen A.Q., Ryu J., Del Mármol J. (2024). Structural basis of odor sensing by insect heteromeric odorant receptors. Science.

[17] Ai D., Dong C., Yang B., Yu C., Wang G. (2022). A fructose receptor gene influences development and feed intake in *Helicoverpa armigera*. Insect Sci..

[18] Yin N.-N., Nuo S.-M., Xiao H.-Y., Zhao Y.-J., Zhu J.-Y., Liu N.-Y. (2021). The ionotropic receptor gene family in Lepidoptera and Trichoptera: Annotation, evolutionary and functional perspectives. Genomics.

[19] Ai M., Min S., Grosjean Y., Leblanc C., Bell R., Benton R., Suh G.S.B. (2010). Acid sensing by the Drosophila olfactory system. Nature.

[20] Silbering A.F., Rytz R., Grosjean Y., Abuin L., Ramdya P., Jefferis G.S., Benton R. (2011). Complementary function and integrated wiring of the evolutionarily distinct *Drosophila* olfactory subsystems. J. Neurosci..

[21] Croset V., Rytz R., Cummins S.F., Budd A., Brawand D., Kaessmann H., Gibson T.J., Benton R. (2010). Ancient protostome origin of chemosensory ionotropic glutamate receptors and the evolution of insect taste and olfaction. PLoS Genet..

[22] Min S., Ai M., Shin S.A., Suh G.S.B. (2013). Dedicated olfactory neurons mediating attraction behavior to ammonia and amines in *Drosophila*. Proc. Natl. Acad. Sci. USA.

[23] Abuin L., Bargeton B., Ulbrich M.H., Isacoff E.Y., Kellenberger S., Benton R. (2011). Functional architecture of olfactory ionotropic glutamate receptors. Neuron.

[24] Budelli G., Ni L., Berciu C., van Giesen L., Knecht Z.A., Chang E.C., Kaminski B., Silbering A.F., Samuel A., Klein M. (2019). Ionotropic receptors specify the morphogenesis of phasic sensors controlling rapid thermal preference in *Drosophila*. Neuron.

[25] Knecht Z.A., Silbering A.F., Ni L., Klein M., Budelli G., Bell R., Abuin L., Ferrer A.J., Samuel A., Benton R. (2016). Distinct combinations of variant ionotropic glutamate receptors mediate thermosensation and hygrosensation in *Drosophila*. eLife.

[26] Enjin A., Zaharieva E.E., Frank D.D., Mansourian S., Suh G.S., Gallio M., Stensmyr M.C. (2016). Humidity sensing in *Drosophila*. Curr. Biol..

[27] Ni L., Klein M., Svec K.V., Budelli G., Chang E.C., Ferrer A.J., Benton R., Samuel A.D.T., Garrity P.A. (2016). The ionotropic receptors IR21a and IR25a mediate cool sensing in *Drosophila*. eLife.

[28] Koh T.W., He Z., Gorur-Shandilya S., Menuz K., Larter N.K., Stewart S., Carlson J.R. (2014). The *Drosophila* IR20a clade of ionotropic receptors are candidate taste and pheromone receptors. Neuron.

[29] Stewart S., Koh T.W., Ghosh A.C., Carlson J.R. (2015). Candidate ionotropic taste receptors in the *Drosophila larva*. Proc. Natl. Acad. Sci. USA.

[30] Tauber J.M., Brown E.B., Li Y., Yurgel M.E., Masek P., Keene A.C. (2017). A subset of sweet-sensing neurons identified by IR56d are necessary and sufficient for fatty acid taste. PLoS Genet..

[31] Olivier V., Monsempes C., François M.-C., Poivet E., Jacquin-Joly E. (2011). Candidate chemosensory ionotropic receptors in a Lepidoptera. Insect Mol. Biol..

[32] Peng Y., Wu S., Hu S., Wang P., Liu T., Fan Y., Wang J., Jiang H. (2024). Ionotropic receptor 8a (Ir8a) plays an important role in acetic acid perception in the oriental fruit fly, *Bactrocera dorsalis*. J. Agric. Food Chem..

[33] Li Z., Zhang Y., An X., Wang Q., Khashaveh A., Gu S., Liu S., Zhang Y. (2020). Identification of leg chemosensory genes and sensilla in *Apolygus lucorum*. Front. Physiol..

[34] Li F., Venthur H., Lin K., Zhang C., Chen Z., Zhou J.-J. (2025). Insect chemosensory proteins as targets in insecticide resistance and development. New Plant Prot..

[35] Cassau S., Krieger J. (2021). The role of SNMPs in insect olfaction. Cell Tissue Res..

[36] Liu Y.-L., Guo H., Huang L.-Q., Pelosi P., Wang C.-Z. (2014). Unique function of a chemosensory protein in the proboscis of two Helicoverpa species. J. Exp. Biol..

[37] Wang H., Zhao R., Gao J., Xiao X., Yin X., Hu S., Zhang Y., Liang P., Gu S. (2024). Two cuticle-enriched chemosensory proteins confer multi-insecticide resistance in *Spodoptera frugiperda*. Int. J. Biol. Macromol..

[38] Wang Y., Dong H., Qu Y., Zhou Y., Qin J., Li K., Luo C., Ren B., Cao Y., Zhang S. (2024). Circabidian rhythm of sex pheromone reception in a scarab beetle. Curr. Biol..

[39] Zhou Y., Zhou L., Li Q., Zhu X., Yu Z., Ke H., Chen Q., Ren B. (2023). Transcriptome analysis and identification of genes related to environmental adaptation of *Grylloprimevala jilina* Zhou & Ren 2023. Ecol. Evol..

[40] Kim D., Langmead B., Salzberg S.L. (2015). HISAT: A fast spliced aligner with low memory requirements. Nat. Methods.

[41] Pertea M., Pertea G.M., Antonescu C.M., Chang T.-C., Mendell J.T., Salzberg S.L. (2015). StringTie enables improved reconstruction of a transcriptome from RNA-seq reads. Nat. Biotechnol..

[42] Buchfink B., Xie C., Huson D.H. (2015). Fast and sensitive protein alignment using DIAMOND. Nat. Methods.

[43] Jones P., Binns D., Chang H.-Y., Fraser M., Li W., McAnulla C., McWilliam H., Maslen J., Mitchell A., Nuka G. (2014). InterProScan 5: Genome-scale protein function classification. Bioinformatics.

[44] Finn R.D., Bateman A., Clements J., Coggill P., Eberhardt R.Y., Eddy S.R., Heger A., Hetherington K., Holm L., Mistry J. (2014). Pfam: The protein families database. Nucleic Acids Res..

[45] Katoh K., Standley D.M. (2013). MAFFT multiple sequence alignment software version 7: Improvements in performance and usability. Mol. Biol. Evol..

[46] Nguyen L.-T., Schmidt H.A., von Haeseler A., Minh B.Q. (2015). IQ-TREE: A fast and effective stochastic algorithm for estimating maximum-likelihood phylogenies. Mol. Biol. Evol..

[47] Kalyaanamoorthy S., Minh B.Q., Wong T.K.F., von Haeseler A., Jermiin L.S. (2017). ModelFinder: Fast model selection for accurate phylogenetic estimates. Nat. Methods.

[48] Hoang D.T., Chernomor O., von Haeseler A., Minh B.Q., Vinh L.S. (2018). UFBoot2: Improving the ultrafast bootstrap approximation. Mol. Biol. Evol..

[49] Ranwez V., Harispe S., Delsuc F., Douzery E.J.P. (2011). MACSE: Multiple alignment of coding sequences accounting for frameshifts and stop codons. PLoS ONE.

[50] Tamura K., Stecher G., Kumar S. (2021). MEGA11: Molecular Evolutionary Genetics Analysis version 11. Mol. Biol. Evol..

[51] Ronquist F., Teslenko M., van der Mark P., Ayres D.L., Darling A., Höhna S., Larget B., Liu L., Suchard M.A., Huelsenbeck J.P. (2012). MrBayes 3.2: Efficient Bayesian phylogenetic inference and model choice across a large model space. Syst. Biol..

[52] Zhang D., Gao F., Jakovlić I., Zou H., Zhang J., Li W.X., Wang G.T. (2020). PhyloSuite: An integrated and scalable desktop platform for streamlined molecular sequence data management and evolutionary phylogenetics studies. Mol. Ecol. Resour..

[53] Letunic I., Bork P. (2021). Interactive Tree Of Life (iTOL) v5: An online tool for phylogenetic tree display and annotation. Nucleic Acids Res..

[54] Li G., Du J., Li Y., Wu J. (2015). Identification of putative olfactory genes from the oriental fruit moth *Grapholita molesta* via an antennal transcriptome analysis. PLoS ONE.

[55] Guo M., Du L., Chen Q., Feng Y., Zhang J., Zhang X., Tian K., Cao S., Huang T., Jacquin-Joly E. (2021). Odorant receptors for detecting flowering plant cues are functionally conserved across moths and butterflies. Mol. Biol. Evol..

[56] Cao D., Liu Y., Wei J., Liao X., Walker W.B., Li J., Wang G. (2014). Identification of candidate olfactory genes in *Chilo suppressalis* by antennal transcriptome analysis. Int. J. Biol. Sci..

[57] Jacquin-Joly E., Legeai F., Montagné N., Monsempes C., François M.-C., Poulain J., Gavory F., Walker W.B., Hansson B.S., Larsson M.C. (2012). Candidate chemosensory genes in female antennae of the noctuid moth *Spodoptera littoralis*. Int. J. Biol. Sci..

[58] Walker W.B., Gonzalez F., Garczynski S.F., Witzgall P. (2016). The chemosensory receptors of codling moth *Cydia pomonella*—Expression in larvae and adults. Sci. Rep..

[59] Yang J., Guo H., Jiang N.-L., Tang R., Li G.-C., Huang L.-Q., van Loon J.J.A., Wang C.-Z. (2021). Identification of a gustatory receptor tuned to sinigrin in the cabbage butterfly *Pieris rapae*. PLoS Genet..

[60] Koenig C., Hirsh A., Bucks S., Klinner C., Vogel H., Shukla A., Mansfield J.H., Morton B., Hansson B.S., Grosse-Wilde E. (2015). A reference gene set for chemosensory receptor genes of *Manduca sexta*. Insect Biochem. Mol. Biol..

[61] Guo H., Cheng T., Chen Z., Jiang L., Guo Y., Liu J., Li S., Taniai K., Asaoka K., Kadono-Okuda K. (2017). Expression map of a complete set of gustatory receptor genes in chemosensory organs of *Bombyx mori*. Insect Biochem. Mol. Biol..

[62] Briscoe A.D., Macias-Muñoz A., Kozak K.M., Walters J.R., Yuan F., Jamie G.A., Martin S.H., Dasmahapatra K.K., Ferguson L.C., Mallet J. (2013). Female behaviour drives expression and evolution of gustatory receptors in butterflies. PLoS Genet..

[63] Xu W., Papanicolaou A., Zhang H.-J., Anderson A. (2016). Expansion of a bitter taste receptor family in the polyphagous insect *Helicoverpa armigera*. Sci. Rep..

[64] Dong J.-F., Yang H.-B., Li D.-X., Yu H.-Q., Tian C.-H. (2023). Identification and expression analysis of chemosensory receptors in the tarsi of fall armyworm, *Spodoptera frugiperda* (J. E. Smith). Front. Physiol..

[65] Zhan S., Merlin C., Boore J.L., Reppert S.M. (2011). The monarch butterfly genome yields insights into long-distance migration. Cell.

[66] Corcoran J.A., Jordan M.D., Thrimawithana A.H., Crowhurst R.N., Newcomb R.D. (2015). The peripheral olfactory repertoire of the lightbrown apple moth, *Epiphyas postvittana*. PLoS ONE.

[67] Li H., Hong X., Zeng F., Bai C. (2023). Identification and expression profiles of olfactory-related genes based on transcriptome analysis in *Plodia interpunctella* (Lepidoptera: Pyralidae). Arch. Insect Biochem. Physiol..

[68] Huang C., Zhang X., He D., Wu Q., Tang R., Xing L., Liu W., Wang W., Liu B., Xi Y. (2021). Comparative Genomics Provide Insights into Function and Evolution of Odorant Binding Proteins in *Cydia pomonella*. Front. Physiol..

[69] Chang X.-Q., Nie X.-P., Zhang Z., Zeng F.-F., Lv L., Zhang S., Wang M.-Q. (2017). De novo analysis of the oriental armyworm *Mythimna separata* antennal transcriptome and expression patterns of odorant-binding proteins. Comp. Biochem. Physiol. Part D Genom. Proteom..

[70] Huang X., Yang J., Zou J., Wen X., Wu T., Tian X., Luo J., Niu Y., Huang X. (2024). Identification of odorant binding protein and chemosensory protein genes in *Protegira songi* (Lepidoptera: Noctuidae) via transcriptome analysis. J. Asia-Pac. Entomol..

[71] Agnihotri A., Liu N., Xu W. (2022). Chemosensory Proteins (CSPs) in the Cotton Bollworm *Helicoverpa armigera*. Insects.

[72] Liu S., Chang H., Liu W., Cui W., Liu Y., Wang Y., Ren B., Wang G. (2020). Essential role for SNMP1 in detection of sex pheromones in *Helicoverpa armigera*. Insect Biochem. Mol. Biol..

[73] Venthur H., Zhou J.-J. (2018). Odorant receptors and odorant-binding proteins as insect pest control targets: A comparative analysis. Front. Physiol..

[74] Li R., Jiang G.-F., Shu X.-H., Wang Y.-Q., Li M.-J. (2020). Identification and Expression Profile Analysis of Chemosensory Genes From the Antennal Transcriptome of Bamboo Locust (*Ceracris kiangsu*). Front. Physiol..

[75] Liu Q., Liu W., Zeng B.-S., Wang G., Hao D., Huang Y. (2017). Deletion of the *Bombyx mori* odorant receptor co-receptor (BmOrco) impairs olfactory sensitivity in silkworms. Insect Biochem. Mol. Biol..

[76] Sakurai T., Nakagawa T., Mitsuno H., Mori H., Endo Y., Tanoue S., Yasukochi Y., Touhara K., Nishioka T. (2004). Identification and functional characterization of a sex pheromone receptor in the silkmoth *Bombyx mori*. Proc. Natl. Acad. Sci. USA.

[77] Zhang D.-D., Löfstedt C. (2015). Moth pheromone receptors: Gene sequences, function, and evolution. Front. Ecol. Evol..

[78] Bengtsson J.M., Gonzalez F., Cattaneo A.M., Montagné N., Walker W.B., Bengtsson M., Anfora G., Ignell R., Jacquin-Joly E., Witzgall P. (2014). A predicted sex pheromone receptor of codling moth *Cydia pomonella* detects the plant volatile pear ester. Front. Ecol. Evol..

[79] Li R.-T., Huang L.-Q., Dong J.-F., Wang C.-Z. (2020). A moth odorant receptor highly expressed in the ovipositor is involved in detecting host-plant volatiles. eLife.

[80] Miyamoto T., Slone J., Song X., Amrein H. (2012). A fructose receptor functions as a nutrient sensor in the *Drosophila* brain. Cell.

[81] Jiang X.-J., Ning C., Guo H., Jia Y.-Y., Huang L.-Q., Qu M.-J., Wang C.-Z. (2015). A gustatory receptor tuned to D-fructose in antennal sensilla chaetica of *Helicoverpa armigera*. Insect Biochem. Mol. Biol..

[82] Xu W., Zhang H.-J., Anderson A. (2012). A sugar gustatory receptor identified from the foregut of cotton bollworm *Helicoverpa armigera*. J. Chem. Ecol..

[83] Sato R. (2024). Molecular Functions and Physiological Roles of Gustatory Receptors of the Silkworm *Bombyx mori*. Int. J. Mol. Sci..

[84] Ning C., Yang K., Xu M., Huang L.-Q., Wang C.-Z. (2016). Functional validation of the carbon dioxide receptor in labial palps of *Helicoverpa armigera* moths. Insect Biochem. Mol. Biol..

[85] Zhang J., Duan S., Wang W., Liu D., Wang Y. (2024). Molecular Basis of CO_2_ Sensing in *Hyphantria cunea*. Int. J. Mol. Sci..

[86] Ai M., Blais S., Park J.-Y., Min S., Neubert T.A., Suh G.S.B. (2013). Ionotropic glutamate receptors IR64a and IR8a form a functional odorant receptor complex in vivo in Drosophila. J. Neurosci..

[87] Raji J.I., Melo N., Castillo J.S., Gonzalez S., Saldana V., Stensmyr M.C., DeGennaro M. (2019). Aedes aegypti mosquitoes detect acidic volatiles found in human odor using the IR8a pathway. Curr. Biol..

[88] Zhang X.-X., Yang B., Sun D.-D., Guo M.-B., Zhang J., Wang G.-R. (2022). Ionotropic receptor 8a is involved in the attraction of *Helicoverpa armigera* to acetic acid. Insect Sci..

[89] Pelosi P., Iovinella I., Felicioli A., Dani F.R. (2014). Soluble proteins of chemical communication: An overview across arthropods. Front. Physiol..

[90] Zheng Z.-C., Li D.-Z., Zhou A., Yi S.-C., Liu H., Wang M.-Q. (2016). Predicted structure of a Minus-C OBP from *Batocera horsfieldi* (Hope) suggests an intermediate structure in evolution of OBPs. Sci. Rep..

[91] Xiao S., Sun J.S., Carlson J.R. (2019). Robust olfactory responses in the absence of odorant binding proteins. eLife.

[92] Tu J., Wang Z., Yang F., Liu H., Qiao G., Zhang A., Wang S. (2024). The female-biased general odorant binding protein 2 of *Semiothisa cinerearia* displays binding affinity for biologically active host plant volatiles. Biology.

[93] Pelosi P., Iovinella I., Zhu J., Wang G., Dani F.R. (2018). Beyond chemoreception: Diverse tasks of soluble olfactory proteins in insects. Biol. Rev..

[94] Yi X., Qi J., Zhou X., Hu M.-Y., Zhong G.-H. (2017). Differential expression of chemosensory-protein genes in midguts in response to diet of *Spodoptera litura*. Sci. Rep..

[95] Khuhro S.A., Yan Q., Liao H., Zhu G.-H., Sun J.-B., Dong S.-L. (2018). Expression profile and functional characterization suggesting the involvement of three chemosensory proteins in perception of host plant volatiles in *Chilo suppressalis*. J. Insect Sci..

[96] Tsouri A., Douris V. (2025). The role of chemosensory proteins in insecticide resistance: A review. Insects.

[97] Cassau S., Krieger J. (2024). Evidence for a role of SNMP2 and antennal support cells in sensillum lymph clearance processes of moth pheromone-responsive sensilla. Insect Biochem. Mol. Biol..

[98] Forstner M., Gohl T., Gondesen I., Raming K., Breer H., Krieger J. (2008). Differential expression of SNMP-1 and SNMP-2 proteins in pheromone-sensitive hairs of moths. Chem. Senses.

