# Supplementary material for: Transcriptome Analysis and Identification of Chemosensory Genes in Leguminivora glycinivorella
Source: Biology (Basel). 2026 Mar 21;15(6):505. doi: 10.3390/biology15060505 (PMC13024613; doi:10.3390/biology15060505)
Supplement: Supplementary file 1 [file biology-15-00505-s001.zip › Supplementary Figures S1–S6.pdf]

Supplementary Figures S1–S6

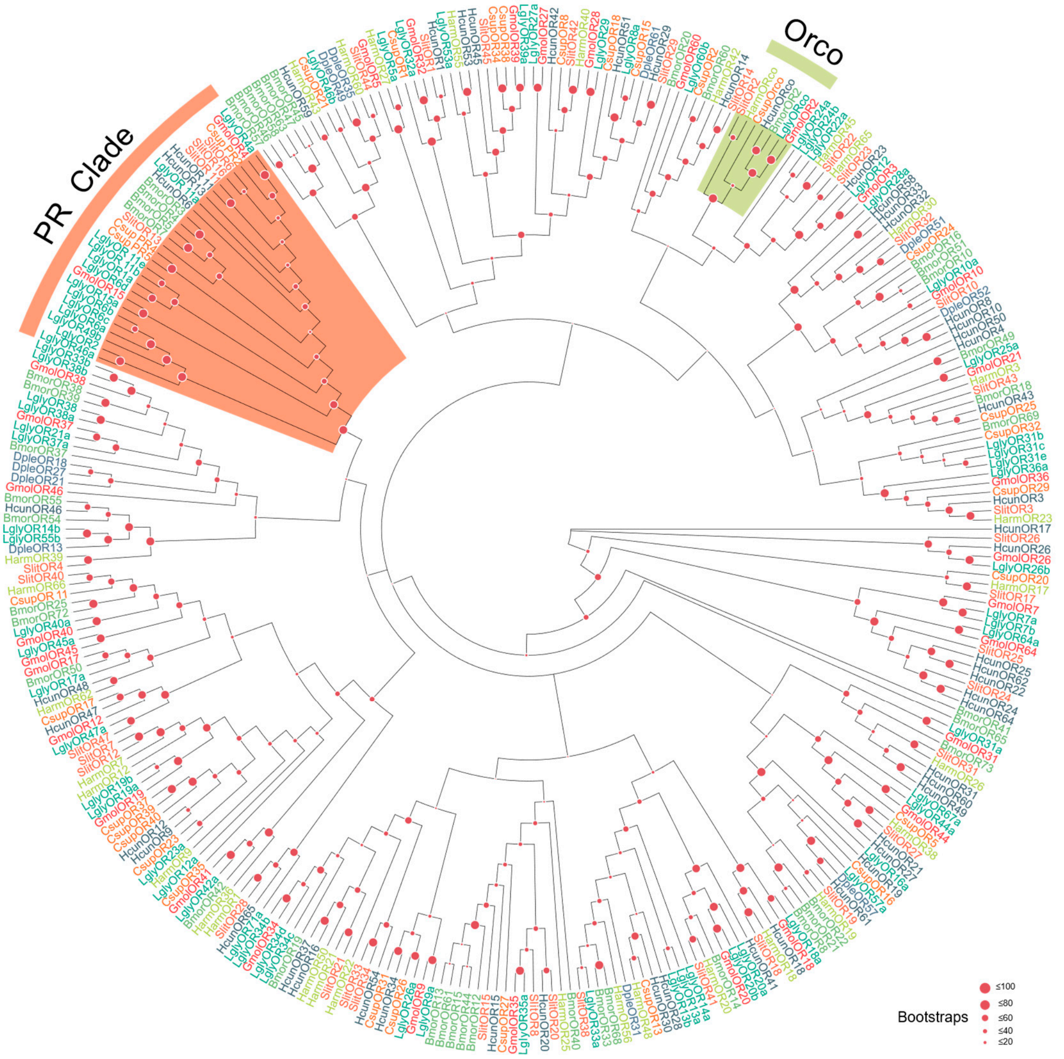

Figure S1. BI phylogenetic tree of ORs from *L. glycinivorella* and representative lepidopteran species based on CDS sequences.

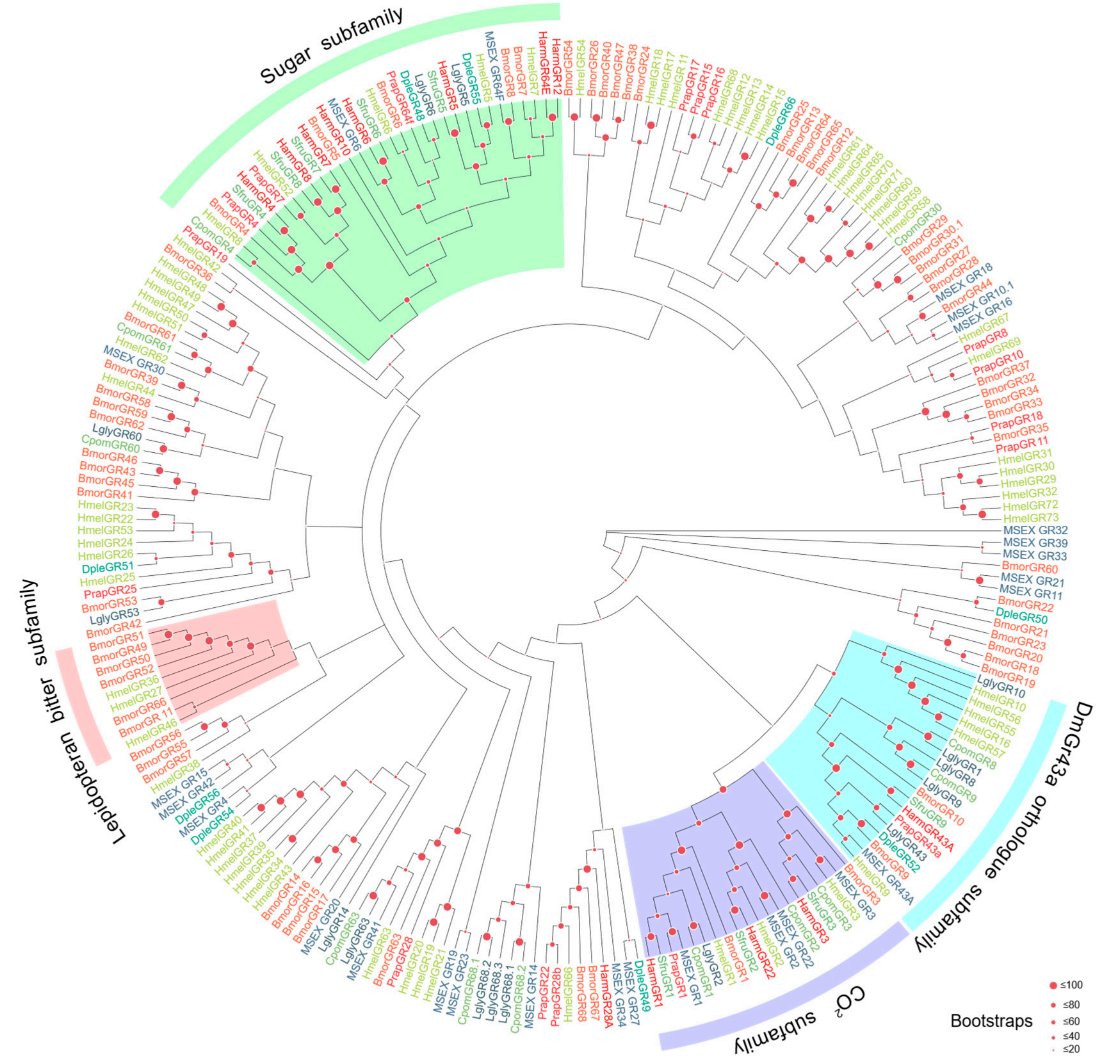

Figure S2. BI phylogenetic tree of GRs from *L. glycinivorella* and representative lepidopteran species based on CDS sequences.

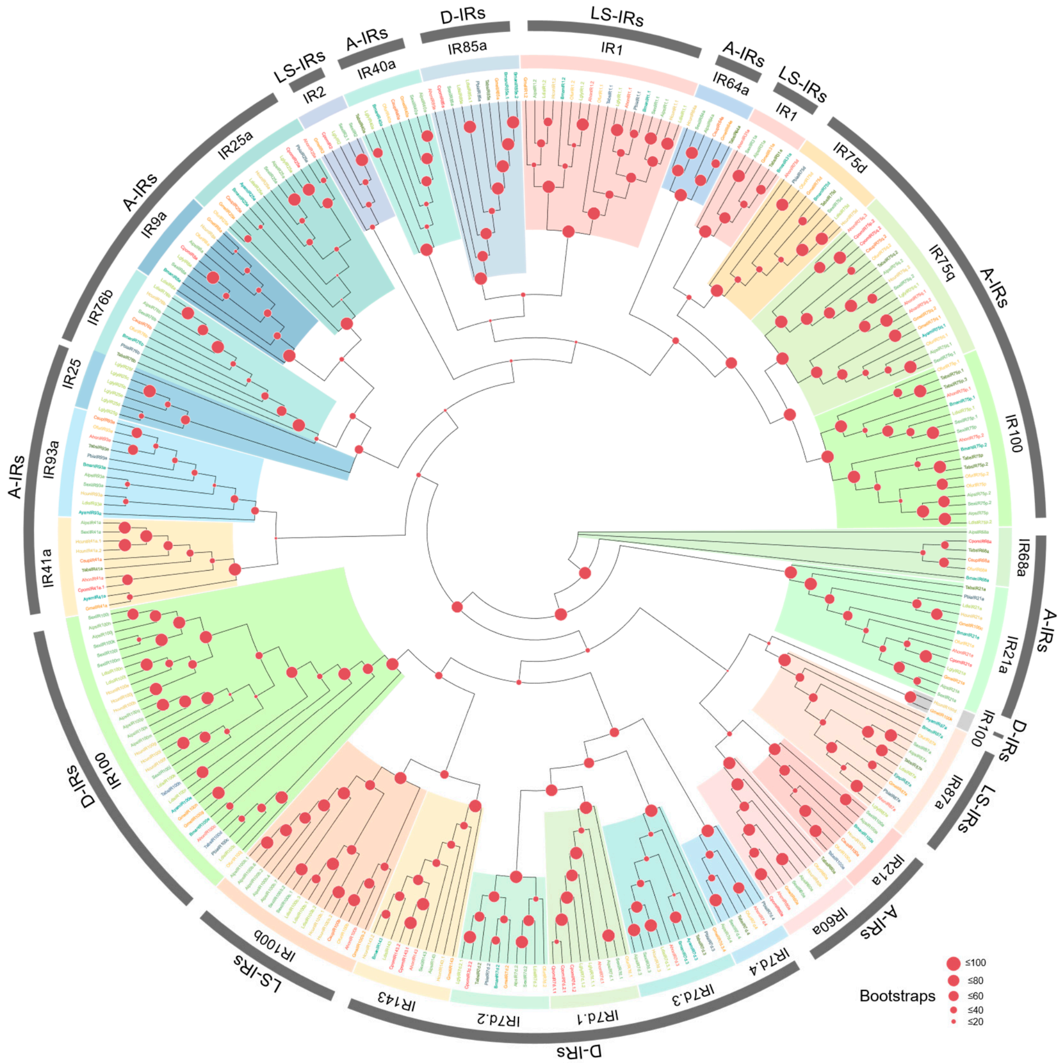

Figure S3. BI phylogenetic tree of IRs from *L. glycinivorella* and representative lepidopteran species based on CDS sequences.

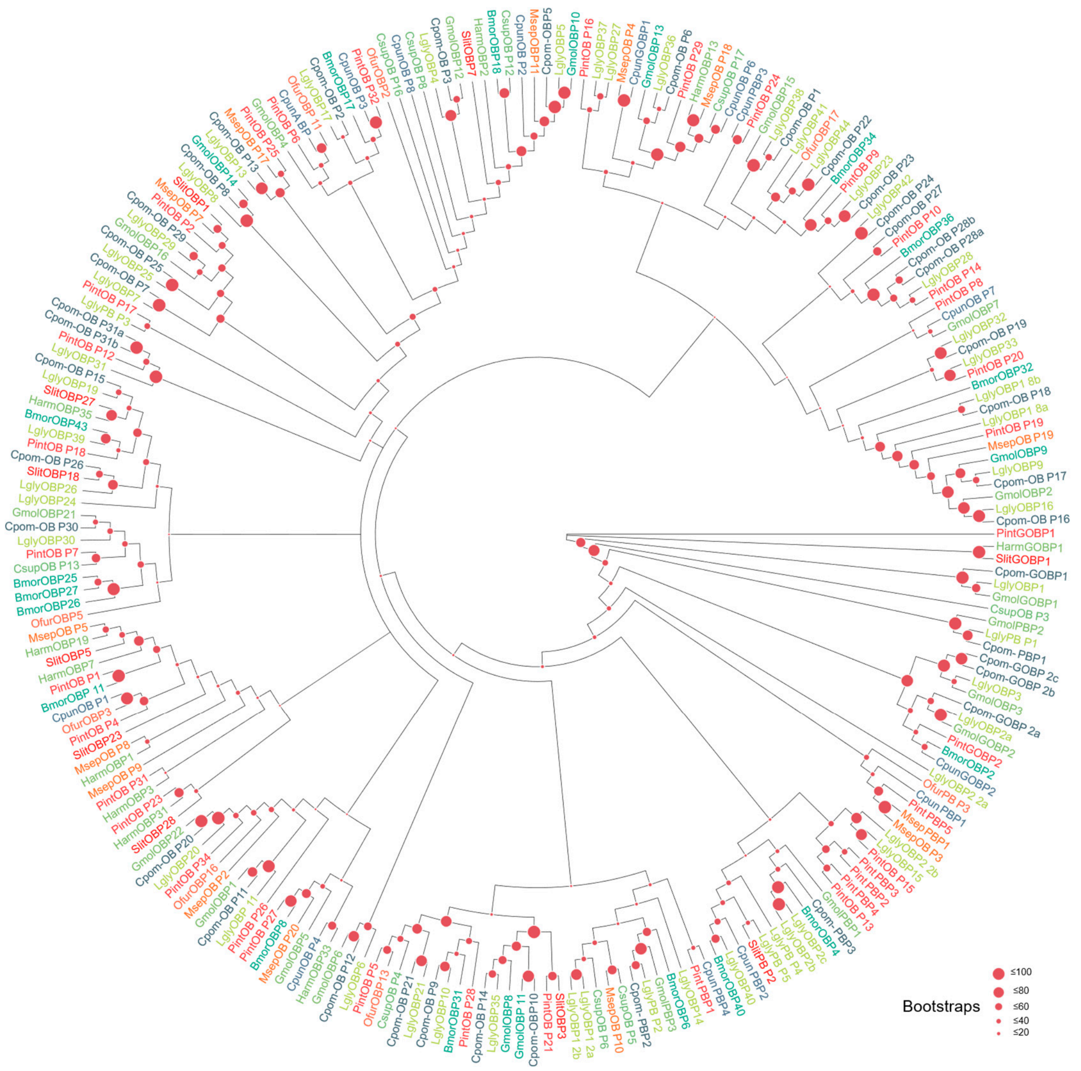

Figure S4. BI phylogenetic tree of OBPs from *L. glycinivorella* and representative lepidopteran species based on CDS sequences.

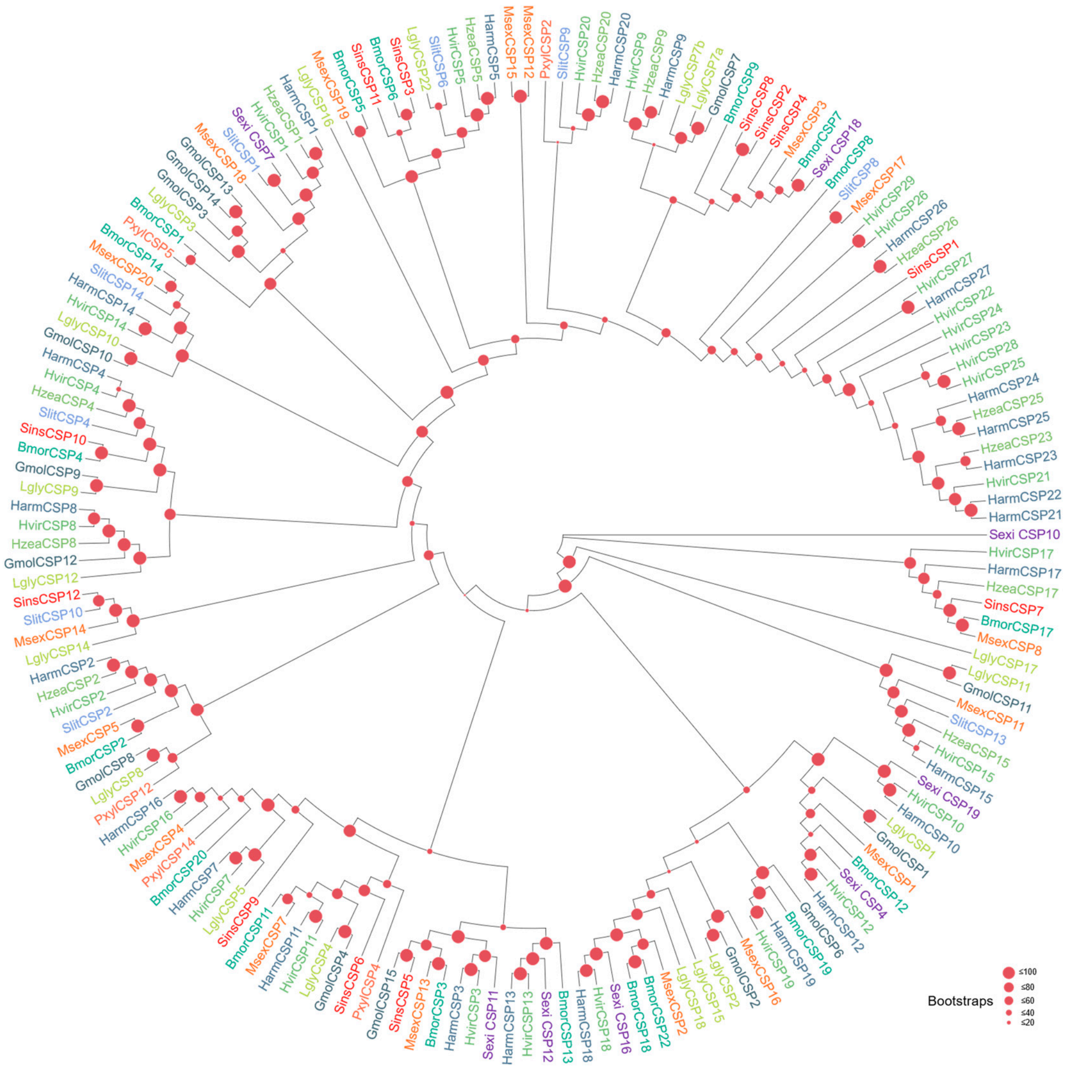

Figure S5. BI phylogenetic tree of CSPs from *L. glycinivorell* and representative lepidopteran species based on CDS sequences.

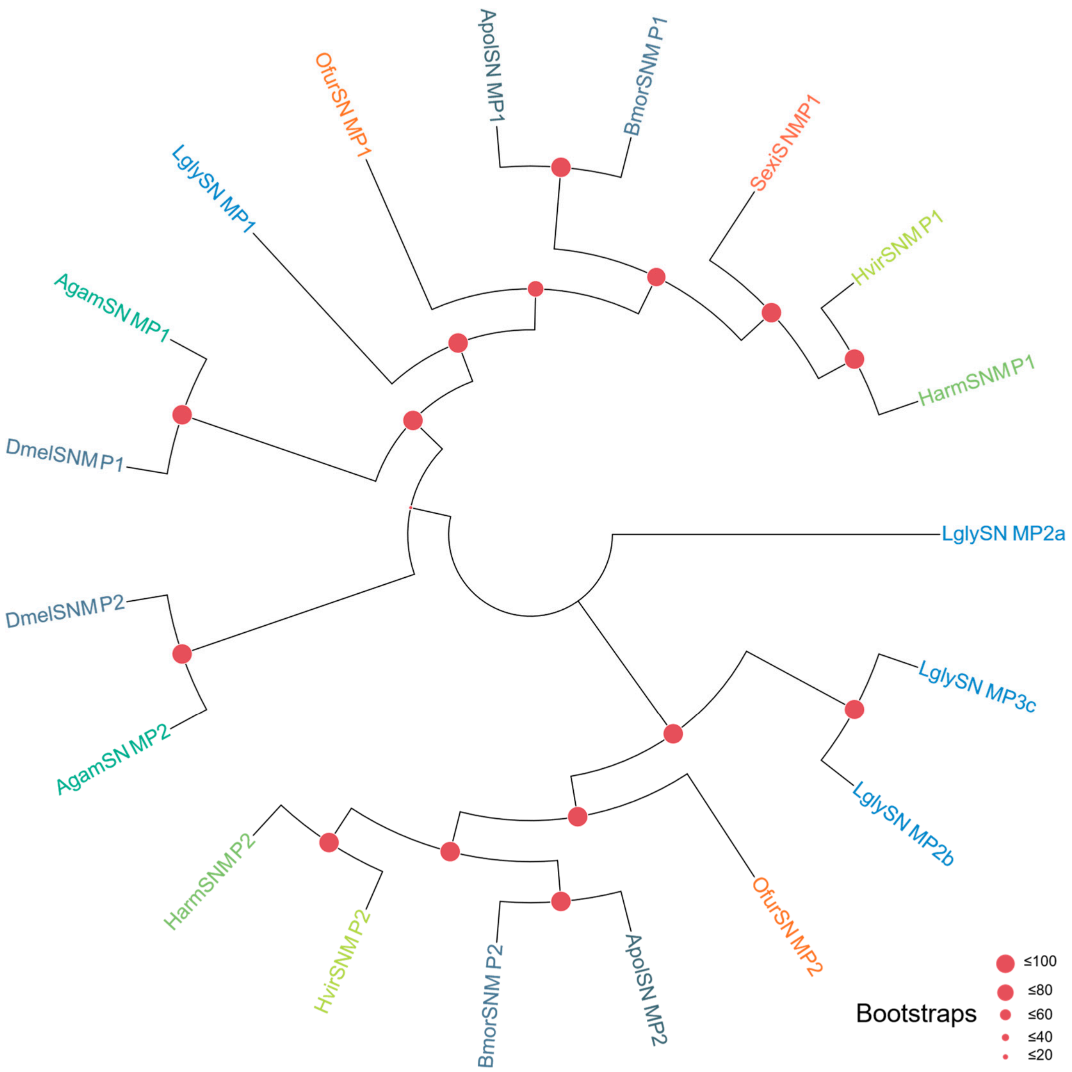

Figure S6. BI phylogenetic tree of SNMPs from *L. glycinivorell* and representative lepidopteran species based on CDS sequences.
