# Supplementary material for: Transcriptome Analysis and Identification of Chemosensory Genes in Leguminivora glycinivorella
Source: Biology (Basel). 2026 Mar 21;15(6):505. doi: 10.3390/biology15060505 (PMC13024613; doi:10.3390/biology15060505)
Supplement: Supplementary file 1 [file biology-15-00505-s001.zip › Table S1 accessions.pdf]

Table S1. BioSample and SRA accession numbers for transcriptome samples of *Leguminivora glycinivorella*

Sample ID	Tissue	Sex	Replicate	BioSample accession (SAMN)	SRA run accession (SRR)
FA-1	Antenna	Female	1	SAMN56395518	SRR37531608
FA-2	Antenna	Female	2	SAMN56395519	SRR37531607
FA-3	Antenna	Female	3	SAMN56395520	SRR37531596
FAb-1	Abdomen	Female	1	SAMN56395521	SRR37531593
FAb-2	Abdomen	Female	2	SAMN56395522	SRR37531592
FAb-3	Abdomen	Female	3	SAMN56395523	SRR37531591
H-1	Head	Female	1	SAMN56395524	SRR37531590
H-2	Head	Female	2	SAMN56395525	SRR37531589
H-3	Head	Female	3	SAMN56395526	SRR37531588
Le-1	Leg	Not determined	1	SAMN56395527	SRR37531587
Le-2	Leg	Not determined	2	SAMN56395528	SRR37531606
Le-3	Leg	Not determined	3	SAMN56395529	SRR37531605
MA-1	Antenna	Male	1	SAMN56395530	SRR37531604
MAb-1	Abdomen	Male	1	SAMN56395531	SRR37531603
MAb-2	Abdomen	Male	2	SAMN56395532	SRR37531602
MAb-3	Abdomen	Male	3	SAMN56395533	SRR37531601
MH-1	Head	Male	1	SAMN56395534	SRR37531600
MH-2	Head	Male	2	SAMN56395535	SRR37531599
MH-3	Head	Male	3	SAMN56395536	SRR37531598
T-1	Thorax	Not determined	1	SAMN56395537	SRR37531597
T-2	Thorax	Not determined	2	SAMN56395538	SRR37531595
T-3	Thorax	Not determined	3	SAMN56395539	SRR37531594

Note. All samples were deposited under BioProject accession number PRJNA1434332.
