# Supplementary material for: Transcriptome Analysis and Identification of Chemosensory Genes in Leguminivora glycinivorella
Source: Biology (Basel). 2026 Mar 21;15(6):505. doi: 10.3390/biology15060505 (PMC13024613; doi:10.3390/biology15060505)
Supplement: Supplementary file 1 [file biology-15-00505-s001.zip › Table S2 qPCR primers.pdf]

Table S2. Primers used for qRT-PCR analysis of selected odorant receptor genes in *Leguminivora glycinivorella*

Gene	Primer	Sequence (5′-3′)	Amplicon size (bp)	Annealing temperature (°C)
LglyOR6a	F	CAGACGCCTTCAGCGAGTAT	113	60
	R	CCCGTACTTGGCCAGTGAAT		60
LglyOR6c	F	ACGGCATGTATTCGGTGGAG	199	60
	R	ATGGCATCAGCTGTGAACGA		60
LglyOR2	F	TCGAAGCTTCTGTGCTGACG	127	60
	R	GTCAGCTCCTCGCTCTTAGTA		60
LglyOR6b	F	AGCACGCCGGCTATTACTAC	157	60
	R	CCCCATATCTGCAGCACCAT		60
LglyOR49b	F	AACGGAATGTTCGGTGACGA	165	60
	R	GTAGGTGAGGATACCGCTGC		60
LglyOR6d	F	GTCACTGGGTGTGTCTTGCT	185	60
	R	TCCATGAGCTCCCAGGGTAA		60
LglyOR1a	F	CGCTGAGGCACTGATACGAT	148	60
	R	ATTGTCCATCTGCTCCCACG		60
LglyOR11b	F	CTGACGGTGTGTTGCTACCT	157	60
	R	TCAACTCGAAAGCCACCGAA		60
LglyORco	F	CCGAACGGGTTGACTCAGAA	100	60
	R	CTATGGACGCCACCAGTCTC		60
LglyOR15a	F	GTTTCACATGGGAGGCCTGA	86	60
	R	TGTACAACAGCAGAGGCAGG		60

Note. F and R indicate forward and reverse primers, respectively. All primer pairs were used with an annealing temperature of 60 °C.
