# Supplementary material for: Transcriptome Analysis and Identification of Chemosensory Genes in Leguminivora glycinivorella
Source: Biology (Basel). 2026 Mar 21;15(6):505. doi: 10.3390/biology15060505 (PMC13024613; doi:10.3390/biology15060505)
Supplement: Supplementary file 1 [file biology-15-00505-s001.zip › Table S3 CSP.pdf]

Table S3. List of candidate CSP genes in *L. glycinivorella*

NO.	Gene name	ID	ORF (aa)	Signal peptide (aa)	BLASTx annotation	Per. Ident	Full length
1	LglyCSP18	gene-LOC125234803	120	1-16	chemosensory protein CSP16 [Lobesia botrana]	86.67%	Yes
2	LglyCSP22	gene-LOC125234789	158	ND	chemosensory protein 10 [Subpsaltria yangi]	43.40%	No
3	LglyCSP12	gene-LOC125234793	203	1-18	chemosensory protein 12 [Grapholita molesta]	77.48%	Yes
4	LglyCSP16	gene-LOC125234415	129	1-17	chemosensory protein 29 [Cnaphalocrocis medinalis]	50.00%	Yes
5	LglyCSP1	gene-LOC125234790	126	1-23	chemosensory protein 1 [Grapholita molesta]	81.51%	Yes
6	LglyCSP17	gene-LOC125234796	131	1-25	chemosensory protein CSP21 [Lobesia botrana]	93.50%	Yes
7	LglyCSP9	gene-LOC125234795	150	1-17	chemosensory protein 9 [Grapholita molesta]	85.71%	Yes
8	LglyCSP8	gene-LOC125232626	119	1-15	chemosensory protein 8 [Grapholita molesta]	84.03%	Yes
9	LglyCSP11	gene-LOC125234797	130	1-24	chemosensory protein 11 [Grapholita molesta]	89.91%	Yes
10	LglyCSP3	gene-LOC125234799	128	1-16	chemosensory protein 3 [Grapholita molesta]	84.50%	Yes
11	LglyCSP2	gene-LOC125234805	107	1-16	chemosensory protein 2 [Grapholita molesta]	55.34%	Yes
12	LglyCSP7a	gene-LOC125234798	128	1-18	chemosensory protein CSP18 [Lobesia botrana]	86.40%	Yes
13	LglyCSP4	gene-LOC125234930	231	1-15	chemosensory protein CSP13 [Cydia pomonella]	91.06%	Yes
14	LglyCSP14	gene-LOC125234523	321	1-16	chemosensory protein CSP20 [Lobesia botrana]	73.60%	Yes
15	LglyCSP10	gene-LOC125234800	127	1-18	chemosensory protein [Grapholita molesta]	89.76%	Yes
16	LglyCSP7b	gene-LOC125234802	125	1-16	chemosensory protein CSP19 [Lobesia	80.00%	Yes

					botrana]		
17	LglyCSP15	gene-LOC125234804	110	1-18	putative chemosensory protein 5 [Conopomorpha sinensis]	28.28%	Yes
18	LglyCSP5	gene-LOC125233847	238	ND	chemosensory protein 14 [Ectropis obliqua]	80.00%	No
