# Supplementary material for: Transcriptome Analysis and Identification of Chemosensory Genes in Leguminivora glycinivorella
Source: Biology (Basel). 2026 Mar 21;15(6):505. doi: 10.3390/biology15060505 (PMC13024613; doi:10.3390/biology15060505)
Supplement: Supplementary file 1 [file biology-15-00505-s001.zip › Table S4 OBP.pdf]

Table S4. List of candidate OBP genes in *L. glycinivorella*

NO.	Gene name	ID	ORF (aa)	Signal peptide (aa)	BLASTx annotation	Per. Ident	Full length
1	LglyOBP1	gene-LOC125234496	169	ND	general odorant-binding protein 1-like isoform X1 [*Leguminivora glycinivorella*]	100.00%	No
2	LglyOBP10	gene-LOC125230027	133	1-16	general odorant-binding protein 56a-like [Cydia strobilella]	96.99%	Yes
3	LglyOBP11	gene-LOC125235538	155	1-22	general odorant-binding protein 69a-like [Leguminivora glycinivorella]	100.00%	Yes
4	LglyOBP12a	gene-LOC125229397	234	1-16	odorant binding protein fmxg18C17 precursor [Bombyx mori]	43.64%	Yes
5	LglyOBP12b	gene-LOC125229396	237	1-19	odorant binding protein fmxg18C17 precursor [Bombyx mori]	42.26%	Yes
6	LglyOBP13	gene-LOC125235211	142	1-21	general odorant-binding protein lush-like [Leguminivora glycinivorella]	100.00%	Yes
7	LglyOBP14	gene-LOC125233539	268	ND	odorant binding proteins OBP11 [Peridroma saucia]	32.54%	No
8	LglyOBP15	gene-LOC125225448	251	1-20	odorant binding protein [Eogystia hippophaecolus]	68.00%	Yes
9	LglyOBP16	gene-LOC125235478	147	1-19	general odorant-binding protein 19d-like [Leguminivora glycinivorella]	100.00%	Yes

10	LglyOBP17	gene-LOC125235209	147	1-26	general odorant-binding protein 72-like [Leguminivora glycinivorella]	100.00%	Yes
11	LglyOBP18a	gene-LOC125235609	128	1-25	general odorant-binding protein 28a-like [Cydia strobilella]	64.41%	Yes
12	LglyOBP18b	gene-LOC125235348	152	1-22	general odorant-binding protein 19d-like [Leguminivora glycinivorella]	100.00%	Yes
13	LglyOBP19	gene-LOC125229734	145	1-16	odorant binding proteins OBP13 [Peridroma saucia]	74.31%	Yes
14	LglyOBP20	gene-LOC125235349	151	1-19	general odorant-binding protein 28a-like [Leguminivora glycinivorella]	100.00%	Yes
15	LglyOBP21	gene-LOC125229694	163	1-28	odorant-binding protein OBP13 [Lobesia botrana]	60.87%	Yes
16	LglyOBP22a	gene-LOC125229638	259	1-19	odorant binding protein [Micromelalopha troglodyta]	47.29%	Yes
17	LglyOBP22b	gene-LOC125229903	257	1-17	odorant binding protein [Athetis dissimilis]	47.68%	Yes
18	LglyOBP23	gene-LOC125236186	176	1-19	general odorant-binding protein 19d-like [Battus philenor]	80.57%	Yes
19	LglyOBP24	NewGene_4510	184	1-19	general odorant-binding protein 68-like [Cydia pomonella]	67.40%	Yes
20	LglyOBP25	gene-LOC125226577	184	1-25	general odorant-binding protein 70 [Leguminivora	100.00%	Yes

					glycinivorella]		
21	LglyOBP26	gene-LOC125229530	198	1-16	odorant-binding protein OBP38 [Lobesia botrana]	69.39%	Yes
22	LglyOBP27	gene-LOC125234795	150	1-17	Putative odorant-binding protein A10 [Papilio machaon]	66.15%	Yes
23	LglyOBP28	gene-LOC125235992	191	ND	general odorant-binding protein 28a [Battus philenor]	71.94%	No
24	LglyOBP29	gene-LOC125234636	240	1-17	odorant binding protein 16 [Grapholita molesta]	87.34%	Yes
25	LglyOBP2a	gene-LOC125234839	160	1-19	general odorant-binding protein 2-like [Leguminivora glycinivorella]	100.00%	Yes
26	LglyOBP2b	gene-LOC125238464	166	1-24	general odorant-binding protein 2-like [Leguminivora glycinivorella]	100.00%	Yes
27	LglyOBP2c	gene-LOC125238470	150	ND	general odorant-binding protein 2-like [Leguminivora glycinivorella]	100.00%	No
28	LglyOBP3	gene-LOC125234842	165	1-21	general odorant-binding protein 2-like [Leguminivora glycinivorella]	100.00%	Yes
29	LglyOBP30	gene-LOC125225334	135	1-16	general odorant-binding protein 56d-like [Leguminivora glycinivorella]	100.00%	Yes
30	LglyOBP31	gene-LOC125230122	335	1-20	odorant binding protein 9 [Spodoptera	55.34%	Yes

					frugiperda]		
31	LglyOBP32	gene-LOC125236004	129	ND	Odorant binding protein [Operophtera brumata]	80.16%	No
32	LglyOBP33	gene-LOC125236002	131	1-15	odorant binding protein 20 [Spodoptera frugiperda]	57.14%	Yes
33	LglyOBP35	gene-LOC125227357	142	1-20	general odorant-binding protein 69a-like [Leguminivora glycinivorella]	100.00%	Yes
34	LglyOBP36	gene-LOC125225663	140	1-17	general odorant-binding protein 83a-like [Leguminivora glycinivorella]	100.00%	Yes
35	LglyOBP37	gene-LOC125235704	149	1-21	odorant binding protein/PBP-general odorant binding protein [Leucinodes orbonalis]	72.34%	Yes
36	LglyOBP38	gene-LOC125236640	178	1-40	odorant binding protein 15 [Grapholita molesta]	87.65%	Yes
37	LglyOBP39	gene-LOC125227135	155	ND	odorant-binding protein OBP39 [Lobesia botrana]	70.51%	No
38	LglyOBP4	gene-LOC125235210	142	1-21	general odorant-binding protein 72-like [Leguminivora glycinivorella]	100.00%	Yes
39	LglyOBP40	gene-LOC125229450	657	1-15	odorant binding protein 26 [Manduca sexta]	49.46%	Yes
40	LglyOBP41	gene-LOC125239867	210	1-18	odorant-binding protein OBP40 [Lobesia botrana]	84.76%	Yes
41	LglyOBP42	gene-LOC125229763	183	1-18	odorant-binding protein OBP35	80.87%	Yes

					[Lobesia botrana]		
42	LglyOBP44	gene-LOC125230505	162	1-16	odorant-binding protein OBP47 [Lobesia botrana]	70.14%	Yes
43	LglyOBP5	gene-LOC125235207	161	ND	general odorant-binding protein 72-like isoform X1 [Leguminivora glycinivorella]	100.00%	No
44	LglyOBP6	gene-LOC125235350	144	1-22	odorant binding protein 6 [Grapholita molesta]	86.81%	Yes
45	LglyOBP7	gene-LOC125232670	137	1-16	general odorant-binding protein 83a-like [Leguminivora glycinivorella]	100.00%	Yes
46	LglyOBP8	gene-LOC125225331	136	1-17	odorant binding protein 14 [Grapholita molesta]	83.09%	Yes
47	LglyOBP9	NewGene11790	146	1-17	odorant-binding protein OBP43 [Lobesia botrana]	71.43%	Yes
48	LglyPBP1	gene-LOC125234837	169	1-25	general odorant-binding protein 1-like [Leguminivora glycinivorella]	100.00%	Yes
49	LglyPBP2	gene-LOC125234841	163	1-21	general odorant-binding protein 1-like [Leguminivora glycinivorella]	100.00%	Yes
50	LglyPBP3	gene-LOC125225284	246	ND	odorant-binding protein OBP48 [Lobesia botrana]	91.85%	No
51	LglyPBP4	gene-LOC125234840	165	1-23	odorant-binding protein PBP1 [Lobesia botrana]	66.88%	Yes
52	LglyPBP5	gene-LOC125240767	164	1-20	odorant-binding protein PBP1	57.23%	Yes
