# Supplementary material for: Transcriptome Analysis and Identification of Chemosensory Genes in Leguminivora glycinivorella
Source: Biology (Basel). 2026 Mar 21;15(6):505. doi: 10.3390/biology15060505 (PMC13024613; doi:10.3390/biology15060505)
Supplement: Supplementary file 1 [file biology-15-00505-s001.zip › Table S5 OR.pdf]

Table S5. List of candidate OR genes in *L. glycinivorell*a

NO.	Gene name	ID	TMD	ORF (aa)	BLASTx annotation	Per. Ident	Full length
1	LglyOR11e	gene-LOC125241650	7	439	putative odorant receptor OR6 [Cydia nigricana]	56.07%	Yes
2	LglyOR21a	gene-LOC125240275	7	409	putative odorant receptor OR56 [Cydia pomonella]	62.21%	No
3	LglyOR38	gene-LOC125229223	6	424	odorant receptors OR38.1 [Lobesia botrana]	64.22%	Yes
4	LglyOR6b	gene-LOC125238744	7	426	putative odorant receptor OR1 [Cydia nigricana]	65.32%	Yes
5	LglyOR29	gene-LOC125232705	5	737	odorant receptor 29 [Hyphantria cunea]	66.92%	No
6	LglyOR34d	gene-LOC125242773	6	397	putative odorant receptor OR55 [Hedya nubiferana]	68.12%	No
7	LglyOR11a	gene-LOC125232706	6	356	putative odorant receptor OR9 [Cydia nigricana]	70.32%	Yes
8	LglyOR57a	gene-LOC125228059	6	396	putative odorant receptor 85d [Amyelois transitella]	71.32%	Yes
9	LglyOR14a	gene-LOC125224837	6	393	odorant receptor 2a-like [Cydia pomonella]	71.51%	Yes
10	LglyOR34c	gene-LOC125242706	6	384	putative odorant receptor OR58 [Cydia pomonella]	72.38%	Yes
11	LglyOR38a	gene-LOC125229224	7	421	odorant receptors OR38.1 [Lobesia botrana]	75.48%	Yes
12	LglyOR2	gene-LOC125241614	7	425	putative odorant receptor OR2 [Cydia nigricana]	77.75%	Yes
13	LglyOR20a	gene-LOC125238837	6	341	odorant receptor 85c-like isoform X1 [Cydia amplana]	79.09%	Yes

14	LglyOR34b	gene-LOC125242705	3	204	putative odorant receptor OR66 [Cydia nigricana]	79.39%	Yes
15	LglyOR34a	gene-LOC125231362	4	262	putative odorant receptor OR66 [Cydia nigricana]	79.39%	Yes
16	LglyOR38b	gene-LOC125231149	7	423	putative odorant receptor OR49 [Cydia pomonella]	81.68%	Yes
17	LglyOR16a	gene-LOC125230521	7	395	putative odorant receptor OR19 [Cydia pomonella]	83.80%	Yes
18	LglyOR37a	gene-LOC125240652	7	379	putative odorant receptor OR56 [Cydia pomonella]	84.96%	No
19	LglyOR12	gene-LOC125228922	6	420	odorant-receptor-12 [Grapholita molesta]	87.38%	Yes
20	LglyOR11b	gene-LOC125236116	7	417	putative odorant receptor OR6 [Cydia fagiglandana]	88.28%	No
21	LglyOR29a	gene-LOC125226314	4	265	odorant receptor 49b-like isoform X1 [Cydia pomonella]	88.30%	Yes
22	LglyOR15a	gene-LOC125228342	7	422	odorant receptor 4-like isoform X1 [Cydia pomonella]	88.44%	Yes
23	LglyOR64a	gene-LOC125224822	6	506	odorant receptor 13a-like [Cydia amplana]	88.52%	No
24	LglyOR14b	gene-LOC125234235	7	386	odorant receptor 10a-like [Cydia pomonella]	88.65%	Yes
25	LglyOR33a	gene-LOC125236719	7	395	odorant receptor 85c-like [Cydia strobilella]	90.38%	Yes
26	LglyOr60b	gene-LOC125231924	7	450	putative odorant receptor OR47 [Cydia fagiglandana]	91.78%	Yes
27	LglyORco	gene-LOC125242219	7	473	odorant receptor coreceptor [Leguminivora glycinivorella]	100.00%	Yes

28	LglyOR9a	gene-LOC125240740	7	395	odorant receptor Or1-like [Leguminivora glycinivorella]	100.00%	No
29	LglyOR8a	gene-LOC125242051	7	402	odorant receptor 13a-like [Leguminivora glycinivorella]	100.00%	Yes
30	LglyOR7b	gene-LOC125242104	7	399	odorant receptor Or1-like [Leguminivora glycinivorella]	100.00%	Yes
31	LglyOR7a	gene-LOC125238311	7	399	odorant receptor Or1-like [Leguminivora glycinivorella]	100.00%	Yes
32	LglyOR71a	gene-LOC125241510	6	357	odorant receptor 4-like [Leguminivora glycinivorella]	100.00%	No
33	LglyOR6d	gene-LOC125238781	7	414	odorant receptor 9a-like [Leguminivora glycinivorella]	100.00%	Yes
34	LglyOR6c	gene-LOC125229176	7	427	putative odorant receptor 92a [Leguminivora glycinivorella]	100.00%	Yes
35	LglyOR6a	gene-LOC125234178	7	425	odorant receptor 43a-like [Leguminivora glycinivorella]	100.00%	Yes
36	LglyOR67a	gene-LOC125242767	7	402	odorant receptor 67a-like [Leguminivora glycinivorella]	100.00%	Yes
37	LglyOR5a	gene-LOC125234112	6	335	odorant receptor 94b-like [Leguminivora glycinivorella]	100.00%	Yes
38	LglyOR55b	gene-LOC125231133	7	383	odorant receptor 10a-like [Leguminivora glycinivorella]	100.00%	Yes
39	LglyOR53a	gene-LOC125228221	7	406	odorant receptor 49b-like	100.00%	No

					[Leguminivora glycinivorella]		
40	LglyOR4a	gene-LOC125241276	6	349	odorant receptor 13a-like [Leguminivora glycinivorella]	100.00%	No
41	LglyOR49b	gene-LOC125241684	7	429	odorant receptor 49b-like [Leguminivora glycinivorella]	100.00%	Yes
42	LglyOR47a	gene-LOC125230276	6	406	odorant receptor 4-like [Leguminivora glycinivorella]	100.00%	No
43	LglyOR46b	gene-LOC125238802	7	393	odorant receptor 46a-like [Leguminivora glycinivorella]	100.00%	No
44	LglyOR46a	gene-LOC125235411	7	332	odorant receptor 46a-like [Leguminivora glycinivorella]	100.00%	No
45	LglyOR45a	gene-LOC125233865	5	273	odorant receptor 67a-like [Leguminivora glycinivorella]	100.00%	No
46	LglyOR44a	gene-LOC125234049	7	432	odorant receptor 46a-like [Leguminivora glycinivorella]	100.00%	Yes
47	LglyOR42a	gene-LOC125230398	7	382	putative odorant receptor 85d [Leguminivora glycinivorella]	100.00%	Yes
48	LglyOR40a	gene-LOC125226384	7	394	odorant receptor 85c-like [Leguminivora glycinivorella]	100.00%	No
49	LglyOR39a	gene-LOC125231298	7	406	odorant receptor 49b-like [Leguminivora glycinivorella]	100.00%	Yes
50	LglyOR36a	gene-LOC125225359	7	411	odorant receptor 13a-like	100.00%	Yes

					[Leguminivora glycinivorella]		
51	LglyOR35a	gene-LOC125234216	3	163	odorant receptor 2a-like [Leguminivora glycinivorella]	100.00%	Yes
52	LglyOR33b	gene-LOC125230745	7	405	odorant receptor 67a-like [Leguminivora glycinivorella]	100.00%	Yes
53	LglyOR32a	gene-LOC125230444	6	445	putative odorant receptor 85e [Leguminivora glycinivorella]	100.00%	No
54	LglyOR31e	gene-LOC125229511	7	428	gustatory and odorant receptor 22-like isoform X1 [Leguminivora glycinivorella]	100.00%	Yes
55	LglyOR31c	gene-LOC125232508	7	424	LOW QUALITY PROTEIN: gustatory and odorant receptor 22-like [Leguminivora glycinivorella]	100.00%	Yes
56	LglyOR31b	gene-LOC125226364	7	511	LOW QUALITY PROTEIN: gustatory and odorant receptor 24 [Leguminivora glycinivorella]	100.00%	No
57	LglyOR31a	gene-LOC125226313	7	400	odorant receptor 46a-like [Leguminivora glycinivorella]	100.00%	No
58	LglyOR28a	gene-LOC125229919	12	759	odorant receptor Or1-like [Leguminivora glycinivorella]	100.00%	Yes
59	LglyOR27a	gene-LOC125225411	7	402	odorant receptor Or2-like [Leguminivora glycinivorella]	100.00%	Yes
60	LglyOR26b	gene-LOC125242238	7	371	odorant receptor 94b-like isoform X1	100.00%	Yes

					[Leguminivora glycinivorella]		
61	LglyOR26a	gene-LOC125227852	7	396	LOW QUALITY PROTEIN: odorant receptor Or1-like [Leguminivora glycinivorella]	100.00%	No
62	LglyOR25a	gene-LOC125242593	7	376	odorant receptor 30a-like [Leguminivora glycinivorella]	100.00%	Yes
63	LglyOR24b	gene-LOC125227313	4	312	odorant receptor 4-like isoform X2 [Leguminivora glycinivorella]	100.00%	Yes
64	LglyOR24a	gene-LOC125239213	5	369	odorant receptor 4-like [Leguminivora glycinivorella]	100.00%	Yes
65	LglyOR23a	gene-LOC125242595	3	172	odorant receptor 67c-like [Leguminivora glycinivorella]	100.00%	No
66	LglyOR22a	gene-LOC125231207	4	247	odorant receptor 4-like [Leguminivora glycinivorella]	100.00%	Yes
67	LglyOR20b	gene-LOC125240316	6	409	odorant receptor 13a-like isoform X2 [Leguminivora glycinivorella]	100.00%	No
68	LglyOR1a	gene-LOC125231872	7	419	putative odorant receptor 92a [Leguminivora glycinivorella]	100.00%	Yes
69	LglyOR19b	gene-LOC125232009	7	401	odorant receptor 4-like [Leguminivora glycinivorella]	100.00%	Yes
70	LglyOR19a	gene-LOC125227753	6	402	putative odorant receptor 92a [Leguminivora glycinivorella]	100.00%	No
71	LglyOR18a	gene-LOC125231605	7	396	odorant receptor 49b-like [Leguminivora glycinivorella]	100.00%	Yes

72	LglyOR17a	gene-LOC125234231	7	407	odorant receptor 13a-like [Leguminivora glycinivorella]	100.00%	Yes
73	LglyOR13b	gene-LOC125224833	7	400	odorant receptor 2a-like [Leguminivora glycinivorella]	100.00%	Yes
74	LglyOR13a	gene-LOC125228640	7	400	odorant receptor 2a-like [Leguminivora glycinivorella]	100.00%	Yes
75	LglyOR12a	gene-LOC125224800	2	188	odorant receptor 85b-like [Leguminivora glycinivorella]	100.00%	Yes
76	LglyOR10a	gene-LOC125232706	7	397	odorant receptor Or1-like [Leguminivora glycinivorella]	100.00%	Yes
