# Supplementary material for: Transcriptome Analysis and Identification of Chemosensory Genes in Leguminivora glycinivorella
Source: Biology (Basel). 2026 Mar 21;15(6):505. doi: 10.3390/biology15060505 (PMC13024613; doi:10.3390/biology15060505)
Supplement: Supplementary file 1 [file biology-15-00505-s001.zip › Table S6 GR.pdf]

Table S6. List of candidate GR genes in *L. glycinivorella*

NO.	Gene name	ID	TMD	ORF (aa)	BLASTx annotation	Per. Ident	Full length
1	LglyGR5	gene-LOC125240879	7	469	gustatory receptor 5a for trehalose-like [Leguminivora glycinivorella]	100.00%	Yes
2	LglyGR6	gene-LOC125241901	8	439	gustatory receptor for sugar taste 64f-like [Leguminivora glycinivorella]	100.00%	Yes
3	LglyGR2	gene-LOC125229511	7	367	LOW QUALITY PROTEIN: gustatory and odorant receptor 22-like [Leguminivora glycinivorella]	99.45%	Yes
4	LglyGR68.1	gene-LOC125230735	6	399	gustatory receptor 68a-like [Leguminivora glycinivorella]	100.00%	Yes
5	LglyGR43	gene-LOC125236445	7	497	gustatory receptor for sugar taste 43a-like [Leguminivora glycinivorella]	100.00%	Yes
6	LglyGR63	gene-LOC125230008	6	420	putative gustatory receptor GR63, partial [Cydia nigricana]	80.00%	Yes
7	LglyGR60	gene-LOC125242060	6	668	putative gustatory receptor GR60, partial [Cydia nigricana]	66.04.%	Yes
8	LglyGR53	gene-LOC125229843	7	416	putative gustatory receptor 2a [Trichoplusia ni]	39.76%	Yes
9	LglyGR8	gene-LOC125236723	6	404	gustatory receptor for sugar taste 43a-like [Cydia strobilella]	73.08.%	Yes

10	LglyGR14	gene-LOC125237308	8	385	gustatory receptor 24, partial [Achelura yunnanensis]	48.41%	Yes
11	LglyGR1	gen7e-LOC125236721	7	440	gustatory receptor for sugar taste 43a-like [Cydia pomonella]	83.93%	Yes
12	LglyGR10	gene-LOC125226532	6	444	gustatory receptor for sugar taste 43a-like [Cydia pomonella]	35.67%	Yes
13	LglyGR68.2	gene-LOC125230085	6	405	gustatory receptor 68a-like [Leguminivora glycinivorella]	100.00%	Yes
14	LglyGR68.3	gene-LOC125230726	6	407	gustatory receptor 8a-like [Leguminivora glycinivorella]	100.00%	Yes
15	LglyGR9	gene-LOC125236709	7	423	gustatory receptor for sugar taste 43a-like [Leguminivora glycinivorella]	100.00%	Yes
