# Supplementary material for: Transcriptome Analysis and Identification of Chemosensory Genes in Leguminivora glycinivorella
Source: Biology (Basel). 2026 Mar 21;15(6):505. doi: 10.3390/biology15060505 (PMC13024613; doi:10.3390/biology15060505)
Supplement: Supplementary file 1 [file biology-15-00505-s001.zip › Table S7 IR.pdf]

Table S7. List of candidate IR genes in *L. glycinivorella*

NO.	Gene name	ID	TMD	ORF (aa)	BLASTx annotation	Per. Ident	Full length
1	LglyIR7d.2.1	gene-LOC125224640	3	583	putative ionotropic receptor IR7d, partial [Cydia pomonella]	62.61%	Yes
2	LglyIR7d.1.1	gene-LOC125224641	3	594	putative ionotropic receptor IR7d, partial [Cydia nigricana]	71.75%	NO
3	LglyIR21a	gene-LOC125225495	3	853	ionotropic receptor 21a [Leguminivora glycinivorella]	100.00 %	Yes
4	LglyIR1.1	gene-LOC125229167	3	636	ionotropic receptor 75a-like [Leguminivora glycinivorella]	99.68%	NO
5	LglyIR87a	gene-LOC125232655	3	519	putative ionotropic receptor IR87a [Cydia fagiglandana]	92.41%	Yes
6	LglyIR1.2	gene-LOC125233256	3	650	ionotropic receptor 75a-like [Leguminivora glycinivorella]	100.00 %	Yes
7	LglyIR2	gene-LOC125235631	3	409	putative ionotropic receptor IR2 [Cydia fagiglandana]	67.33%	Yes
8	LglyIR75q.1	gene-LOC125238225	3	642	ionotropic receptor 75a-like [Leguminivora glycinivorella]	100.00 %	Yes
9	LglyIR8a	gene-LOC125240135	3	860	ionotropic receptor 25a [Leguminivora glycinivorella]	100.00 %	Yes
10	LglyIR25g	gene-LOC125239831	3	752	ionotropic receptor 14, partial [Diaphania glauculalis]	87.12%	Yes
11	LglyIR25a	gene-LOC125229560	3	922	ionotropic receptor 25a [Plodia interpunctella]	91.53%	Yes
12	LglyIR25f	gene-LOC125240991	3	852	ionotropic receptor 2 [Diaphorina citri]	50.12%	Yes
13	LglyIR25c	gene-LOC125229326	3	867	ionotropic receptor 21 [Propsilocerus akamusi]	78.10%	Yes
14	LglyIR25b	gene-LOC125229327	3	679	ionotropic receptor 21 [Propsilocerus akamusi]	77.59%	Yes
15	LglyIR7d.1.2	gene-LOC125242558	3	575	ionotropic receptor 7d.1, partial [Peridroma saucia]	48.76%	Yes
16	LglyIR25d	gene-LOC125229866	3	925	ionotropic receptor 2, partial [Peridroma saucia]	75.09%	Yes
17	LglyIR40a	gene-LOC125226596	3	705	ionotropic receptor 40a [Cydia strobilella]	93.19%	Yes
18	LglyIR25e	gene-LOC125229931	3	829	ionotropic receptor [Endoclita signifer]	38.67%	Yes
