# Supplementary material for: Transcriptome Analysis and Identification of Chemosensory Genes in Leguminivora glycinivorella
Source: Biology (Basel). 2026 Mar 21;15(6):505. doi: 10.3390/biology15060505 (PMC13024613; doi:10.3390/biology15060505)
Supplement: Supplementary file 1 [file biology-15-00505-s001.zip › Table S8 SNMP.pdf]

Table S8. List of candidate SNMP genes in *L. glycinivorella*

NO.	Gene name	ID	ORF (aa)	Signal peptide (aa)	BLASTx annotation	Per. Ident	Full length
1	LglySNMP1	gene-LOC125235515	521	2	PREDICTED: Leguminivora glycinivorella sensory neuron membrane protein 1-like (LOC125235515), mRNA	100.00%	Yes
2	LglySNMP3c	gene-LOC125228739	514	2	PREDICTED: Leguminivora glycinivorella sensory neuron membrane protein 2-like (LOC125228739), mRNA	99.81%	Yes
3	LglySNMP2b	gene-LOC125228975	518	2	PREDICTED: Leguminivora glycinivorella sensory neuron membrane protein 2-like (LOC125228975), mRNA	100.00%	Yes
4	LglySNMP2a	gene-LOC125241756	509	2	PREDICTED: Leguminivora glycinivorella sensory neuron membrane protein 2 (LOC125241756), transcript variant X1, mRNA	100.00%	Yes
